# Polyoxometalate as Hydrogen–Electron Hub in Metal–Organic Complex for Electrocatalytic Nitrate Reduction to Ammonia

**DOI:** 10.1002/advs.202520543

**Published:** 2025-12-22

**Authors:** Qiushuang Jiang, Xinming Wang, Shengji Tian, Haijun Pang, Yongbin Song, Huiyuan Ma

**Affiliations:** ^1^ School of Materials Science and Chemical Engineering Harbin University of Science and Technology Harbin P. R. China; ^2^ School of Chemistry and Chemical Engineering Harbin Institute of Technology Harbin P. R. China

**Keywords:** ammonia synthesis, electrocatalyst, metal‐organic multinuclear complexes, nitrate reduction, polyoxometalates

## Abstract

Electrocatalytic nitrate reduction to ammonia (ENRA) is a sustainable strategy for decentralized ammonia synthesis and wastewater purification, but its efficiency in neutral media is limited by slow water dissociation and insufficient active hydrogen (*H) supply. Herein, we address this challenge through the rational design of chain‐like polyoxometalate‐based metal–organic complexes that feature dinuclear ([M(H_2_O)_2_C_20_O_2_N_4_H_11_]_4_[SiW^VI^_12_O_40_], M‐SiW_12_‐1D) and trinuclear ([M_1.5_(H_2_O)_2_C_20_O_3_N_4_H_11_]_4_[PW^VI^_7_W^V^_5_O_40_], M‐PW_12_‐1D) architectures (M = Ni, Co, Fe) that create continuous electron channels. Among them, trinuclear Ni‐PW_12_‐1D exhibits exceptional ENRA performance in neutral electrolytes, delivering a very high Faradaic efficiency of 95.0% and a remarkable ammonia yield rate of 16.9 mg h^−1^ mg_cat._^−1^ at −1.2 V vs. RHE. In situ Fourier‐transform infrared spectroscopy, differential electrochemical mass spectrometry, and density functional theory calculations are used to elucidate the synergistic mechanism operating in this system: polyoxometalate (POM) moieties promote water dissociation to generate *H that migrates to metal–organic reaction sites. Meanwhile, electron transfer from the POM to the metal cluster enhances the adsorption of the nitrate intermediate and lowers the energy barrier of the rate‐determining step (NO → *NOH). This synergy balances hydrogenation and suppresses the competing hydrogen evolution reaction, providing insights into multinuclear cluster structure–activity relationships for sustainable nitrogen cycling.

## Graphical abstract

To tackle low electrocatalytic nitrate reduction to ammonia efficiency in neutral media, this work designs chain‐like dinuclear/trinuclear polyoxometalate‐based metal‐organic complexes. Metal clusters provide nitrate adsorption/activation sites, while polyoxometalates (POMs) drive water dissociation to generate active hydrogen (*H) that migrates to these sites; POM‐to‐cluster electron transfer optimizes adsorption and lowers the rate‐determining step barrier to advance sustainable nitrogen cycling.

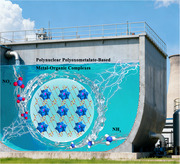

## 1 Introduction

Ammonia (NH_3_) is an indispensable chemical in modern society; however, its industrial synthesis still predominantly relies on the energy‐intensive Haber–Bosch process [1, 2, 3], which requires extreme operating conditions (400–500°C, 19–25 MPa), accounts for 1%–2% of global energy consumption, and releases approximately 1.9 metric tons of carbon dioxide (CO_2_) per ton of NH_3_ produced. These drawbacks underscore the importance of developing sustainable and environmentally friendly alternatives [4]. Among emerging strategies, electrocatalytic c reduction to ammonia (ENRA) is gaining increasing levels of attention because it uses nitrate‐rich wastewater as the nitrogen source and exploits the high solubility of nitrate (NO_3_^−^) and the relatively low dissociation energy of the N═O bond [5, 6, 7]. Notably, most nitrate‐laden wastewater exhibits circumneutral pH in which sluggish water‐dissociation kinetics leads to an inadequate supply of active hydrogen (*H) that, in turn, gives rise to issues including poor efficiency, low current densities, and poor NH_3_ selectivity [8,9]. Consequently, developing efficient electrocatalysts capable of balancing *H generation and converting intermediates is critical for advancing the practical use of ENRA.

To date, substantial effort has been dedicated to developing novel catalysts that enhance the efficiency and selectivity of the ENRA process, including bimetallic alloys [10, 11], metal oxides [12], and metal phosphides [13, 14]. Although noble metals and Cu‐based systems have received extensive attention for their remarkable ENRA performance [15, 16, 17], the research landscape is somewhat imbalanced. Consequently, Earth‐abundant metals such as nickel (Ni), cobalt (Co), and iron (Fe) have been the used relatively sparingly despite their compelling cost‐effectiveness and tunabilities [18, 19]. The widespread perception that these metals are unsuitable stems from their high activities toward the hydrogen evolution reaction (HER), which is typically viewed as the dominant competing pathway. However, ENRA is highly dependent on *H, which is generated by splitting H_2_O under neutral conditions [20, 21]. Water splitting fails to supply sufficient *H for ENRA when overly suppressed, ultimately resulting in low a NH_3_‐synthesis efficiency. To address this issue, a strategy in which motifs that ensure sufficient availability of *H while avoiding excessive *H generation are incorporated is required, which in turn suppresses the HER and delivers a high NH_3_‐production rate [22, 23, 24]. For example, the Pd sites in a ternary NiPdP catalyst facilitate the generation and spillover of active hydrogen species (*H) to hydrogen‐deficient Ni_2_P sites while simultaneously suppressing the competing HER, thereby delivering a high NH_3_ yield rate of 0.908 mmol h^−1^ mg_cat._^−1^ and a Faradaic efficiency of 92.6% [25]. Moreover, the Cu_2_O/Cu/Mn_2_O_3_ heterojunction enhances the hydrogen spillover effect, which significantly lowers the energy barrier for *H migration [26]. These cases reveal that optimizing the reaction pathway by leveraging multicomponent synergy is a robust strategy for the development of high‐performance electrocatalysts.

Transition metal phthalocyanines (TMPcs) and analogous metal–organic complexes have emerged as competitive ENRA candidates [27, 28, 29]. Specifically, Adalder et al. solvothermally synthesized β‐phase manganese phthalocyanine (β‐MnPc) and used a 95 mT external magnetic field to induce spin polarization in β‐MnPc, thereby reducing the energy barrier of the rate‐determining *NO‐protonation step from 1.08 to 0.51 eV, while suppressing the HER and ultimately doubling the ammonia‐yield rate (16603.4 µg h^−1^ mg_cat._^−1^) with a Faradaic efficiency of 92.9% at −0.9 V vs. RHE [30]. Unfortunately, single‐component systems such as the TMPcs often lack sufficient synergy between *H supply and intermediate conversion, despite the abovementioned progress, which limits further performance improvements.

Polyoxometalate‐based metal–organic complexes (POMOCs) have emerged as a highly promising class of ENRA electrocatalyst that leverages the unique properties of polyoxometalates (POMs), which are anionic molecular metal oxide clusters with well‐defined structures that are capable of reversibly storing and releasing electrons akin to “electron sponges.” [31] This intrinsic multielectron redox capability enables POMs to continuously replenish protons under negative potentials, thereby promoting the formation of active hydrogen species (*H) via the proton‐coupled electron transfer (PCET) mechanism [32]. For instance, trilacunary Keggin‐type POMs (e.g., SiW_9_) anchored on Pt nanoparticles supported by carbon nanotubes function as efficient proton pumps that enhance proton transfer through PCET, thereby delivering stable performance in proton exchange membrane water electrolyzers [33]. POMs also significantly enhance electron transfer in other scenarios, with the POM/MXene heterostructure (e.g., NMo_6_‐Tris@MXC, where N = Fe, Co, Ni) a notable example in which covalent integration forms a synergistic interface that boosts electron transfer and NO_3_^−^ adsorption while suppressing the HER, leading to a 98.7% Faradaic efficiency and a high NH_3_ yield of 4.99 mg h^−1^ mg_cat._^−1^ [34]. Therefore, the use of POMOCs is an effective strategy for addressing ENRA challenges, as these complexes combine excellent structural stability, inherent POM redox activity, and the catalytic functionalities of metal–organic complexes (MOCs) [35, 36]. However, the mechanism responsible for interfacial synergy between the POM and MOC active sites remains unclear, and the ability to efficiently integrate POMOCs into electrocatalytic systems remains a critical area that requires urgent breakthroughs.

Motivated by the possibilities presented above, we designed a series of chain‐like polynuclear POMOC‐based ENRA catalysts, including dinuclear [M(H_2_O)_2_C_20_O_2_N_4_H_11_]_4_[SiW^VI^_12_O_40_] (M‐SiW_12_‐1D, M = Ni, Co, Fe) and trinuclear [M_1.5_(H_2_O)_2_C_20_O_3_N_4_H_11_]_4_[PW^VI^_7_W^V^_5_O_40_] (M‐PW_12_‐1D, M = Ni, Co, Fe). These materials were constructed based on two key rationales: (i) establishing continuous electron transport channels between the POMs and MOCs to improve interfacial electron mobility, and (ii) leveraging the PCET capabilities of POMs in order to regulate the generation of active hydrogen species (*H) that are then efficiently transported to the ENRA active sites. This dual strategy ensures an adequate supply of atomic hydrogen for hydrogenating key nitrogenous intermediates (e.g., *NO_2_ and *NO), thereby promoting NH_3_ synthesis. Among the synthesized catalysts, trinuclear Ni‐PW_12_‐1D delivered outstanding ENRA performance, with a Faradaic efficiency of 95.0% and an NH_3_ yield rate of 16.9 mg h^−1^ mg_cat._^−1^ at −1.2 V vs. RHE. The combination of experimental and density functional theory (DFT) studies revealed the pronounced synergy that exists between the POMs and MOCs in Ni‐PW_12_‐1D; this synergy renders the structure of such multinuclear POMOCs favorable for accelerating the rate‐determining step (*NO → *NOH) while enabling the efficient generation of *H, thereby facilitating the efficient electrochemical synthesis of NH_3_ in neutral media. This study provides valuable perspectives for the rational design of high‐performance multinuclear POMOC‐based ENRA electrocatalysts.

## 2 Results and Discussion

### 2.1 Materials Characterization

**FIGURE 1:**
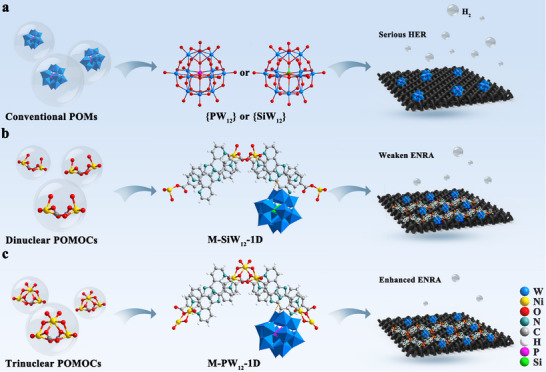
Comparative schematic illustrating the modulation of different POM‐based compounds on HER and ENRA. (a) Conventional polyoxometalates PW_12_ or SiW_12_, (b) Ni‐SiW_12_‐1D, (c) Ni‐PW_12_‐1D.

A POM is prone to severe HER during catalysis in an ENRA system owing to its intrinsic structural and electronic characteristics. The highly reactive HER competes with ENRA for protons and electrons, which substantially diminishes ammonia production efficiency (Figure 1a). The M‐SiW_12_‐1D materials fabricated from the dinuclear POMOCs were found to exert a weak promotional effect on the ENRA process, failing to efficiently drive nitrate reduction to ammonia; consequently, their catalytic performance is unable to satisfy ENRA demands (Figure 1b). In contrast, the M‐PW_12_‐1D materials constructed from the trinuclear POMOCs efficiently adsorbed and activated nitrate while inhibiting side reactions such as the HER owing to their abundant active sites, matched electronic structures, and spatial configurations, thereby remarkably enhancing the ENRA process and delivering highly efficient ammonia synthesis (Figure 1c).

The crystal structures of the Ni‐PW_12_‐1D, Ni‐SiW_12_‐1D, and Co‐SiW_12_‐1D materials were determined by single‐crystal X‐ray diffractometry (SCXRD). The phase purities and structural consistencies of the remaining isostructural analogs (Co‐PW_12_‐1D, Fe‐PW_12_‐1D, and Fe‐SiW_12_‐1D) were verified by the close agreement observed between their experimental and simulated powder X‐ray diffraction (PXRD) patterns. SCXRD revealed that Ni‐PW_12_‐1D crystallizes in the monoclinic C2/m space group. Figure S1 and Tables S1 and S2 show that the asymmetric unit of Ni‐PW_12_‐1D contains 1/4 of a {PW_12_O_40_} cluster, 1.5 Ni^2+^ ions, and one HNCP ligand. Ni^2+^ ions are present in two coordination environments: Ni(1) shows a six‐coordinate geometry and is coordinated by two oxygen atoms (O(3) and O(4)) from coordinated H_2_O molecules, along with two nitrogen atoms (N(3) and N(4)) and two oxygen atoms (O(1) and O(2)) from an HNCP ligand. In contrast, Ni(2) resides in a typical octahedral configuration and is coordinated by four oxygen atoms from water molecules (O(2), O(4)) and two oxygen atoms (O(5)) derived from HNCP ligands. Additionally, the {PW_12_O_40_} cluster engages in hydrogen bonding between the N(1) atom of an HNCP ligand and the terminal oxygen O(13) of the {PW_12_O_40_} anion (d(N(1)⋅⋅⋅O(13)) = 2.851 Å). These 1D chain‐like structural units are further assembled into a highly ordered 3D supramolecular framework through π‐π stacking interactions and intermolecular forces, and display prominent structural regularity.

Ni‐SiW_12_‐1D and Co‐SiW_12_‐1D exhibit analogous primary structures, with their central transition metal ions (Ni^2+^ and Co^2+^, respectively) as the sole distinction (Figure S2 and Tables S1, S3, and S4). Both compounds crystallized in the monoclinic C2/m space group, with asymmetric structural units comprising 1/4 of a {SiW_12_O_40_} cluster, one central {M} ion (M = Ni^2+^ or Co^2+^), and one HNCP ligand. Taking Ni‐SiW_12_‐1D as a representative example, the Ni(1) center adopts a distorted octahedral geometry coordinated by two water‐derived oxygen atoms (O(1) and O(2)), two nitrogen atoms (N(1) and N(2)), and two oxygen atoms (O(3) and O(4)) from HNCP ligands, to form a 1D chain. Additionally, the {SiW_12_O_40_} cluster engages in hydrogen‐bonding interactions with adjacent HNCP ligands, specifically between the N(3) atom of the ligand and the terminal oxygen O(5) of the {SiW_12_O_40_} anion (d(N(3)⋅⋅⋅O(5)) = 2.881 Å). These 1D chain‐like structural units are further assembled into a highly ordered 3D supramolecular framework through π‐π stacking interactions and intermolecular forces, and display prominent structural regularity.

The microstructures of the as‐synthesized M‐PW_12_‐1D and M‐SiW_12_‐1D were examined by scanning electron microscopy (SEM) (Figures S3–S8), which revealed well‐defined rectangular prismoidal morphologies with lengths ranging from approximately 80 to 150 µm. Elemental mapping confirmed that the key constituent elements (e.g., M = Ni, Co, or Fe; C, N, O, P/Si, W) are homogeneously distributed throughout each microstructure. The PXRD patterns of both series of samples are in good agreement with the simulations generated from their corresponding single‐crystal structures (Figure S9,b), attesting to the high phase purity and structural integrity of the prepared compounds. The almost identical diffraction profiles observed within the M‐PW_12_‐1D and M‐SiW_12_‐1D (Ni, Co, Fe) groups are noteworthy as they provide strong evidence of their isostructural nature and corroborate the structural models established by single‐crystal analysis.

The Fourier transform infrared (FTIR) spectra of the M‐PW_12_‐1D and M‐SiW_12_‐1D series confirmed that the POMs and organic components had been successfully integrated (Figure S10a,b). Specifically, both sets of samples exhibited bands at 798, 889–921, 981–984, 1080, and 1018 cm^−1^ that are assigned to W‐O_c_‐W, W‐O_b_‐W, W = O_d_, P‐O_a_ and Si‐O_a_ stretching vibrations, respectively [37]. Additionally, the absorptions observed between 1230 and 1710 cm^−1^ confirm that the HNCP ligand had been successful incorporated into each material [38]. Furthermore, M‐PW_12_‐1D and M‐SiW_12_‐1D exhibited absorption peaks at 642 and 645 cm^−1^, respectively, which are attributable to M‐O stretching vibrations [39].

**FIGURE 2:**
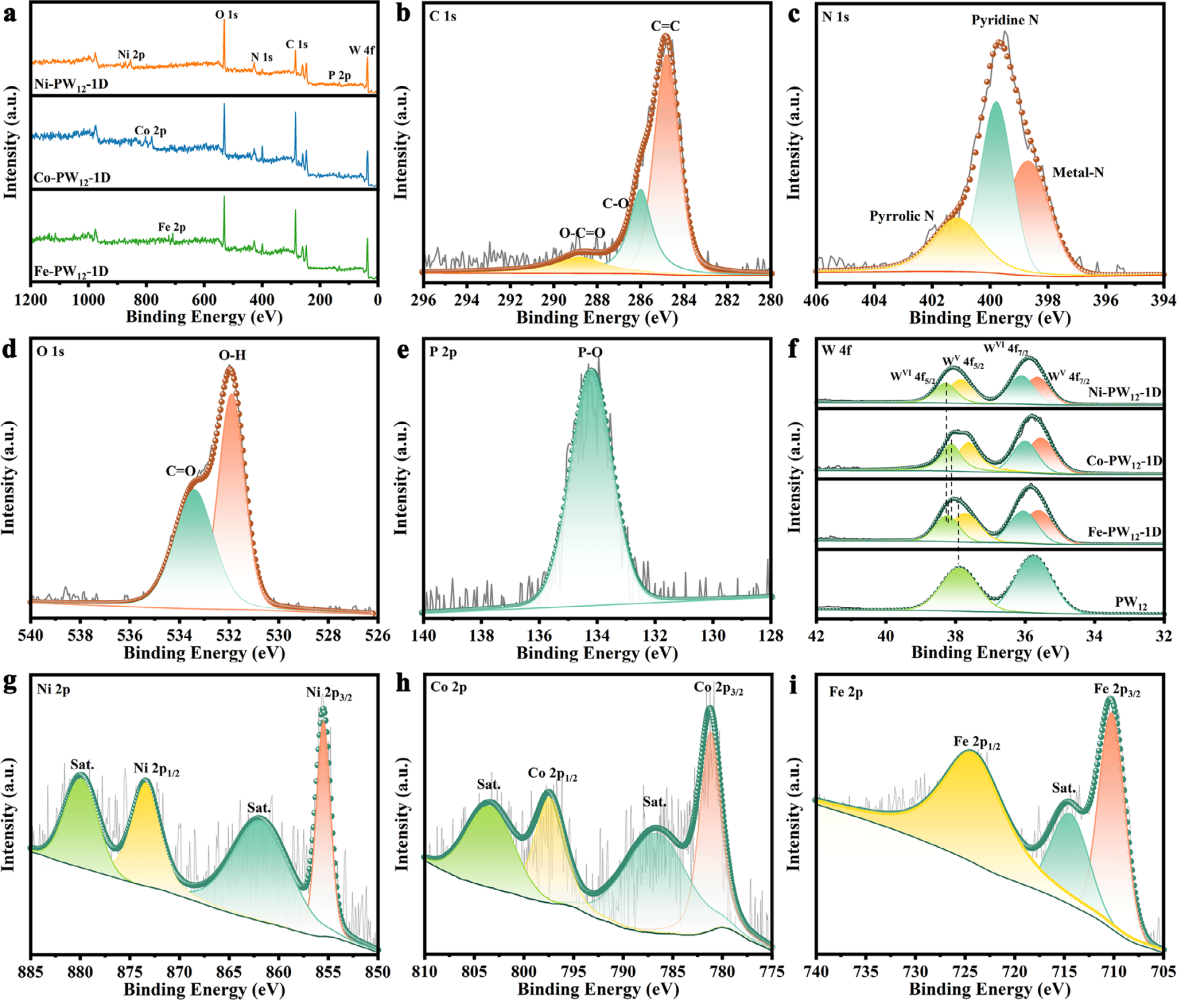
XPS spectra of Ni‐PW_12_‐1D: (a) survey, (b) C 1s, (c) N 1s, (d) O 1s and (e) P 2p. (f) W 4f, (g) Ni 2p, (h) Co 2p and (i) Fe 2p spectra of PW_12_ and M‐PW_12_‐1D.

The chemical states and compositions of the synthesized catalysts were examined by X‐ray photoelectron spectroscopy (XPS). The survey spectra presented in Figure 2a and Figure S11a confirm that M‐PW_12_‐1D and M‐SiW_12_‐1D are composed of C, N, O, P/Si, W, Ni, Co, and Fe. The C 1s spectra of Ni‐PW_12_‐1D and Ni‐SiW_12_‐1D exhibit characteristic C═C (284.8 eV), C─O (286.0 eV), and O─C═O (288.8 eV) peaks (Figure 2b; Figure S11b) [40]. The N 1s spectra were deconvoluted (Figure 2c; Figure S11c), which revealed that Ni‐PW_12_‐1D and Ni‐SiW_12_‐1D contain pyrrolic N (401.1 and 401.3 eV), pyridinic N (399.8 and 399.9 eV), and metal‐N (398.7 eV) [41]. The O 1s spectra (Figure 2d: Figure S11d) of Ni‐PW_12_‐1D and Ni‐SiW_12_‐1D show doublets at 531.9 and 533.4 eV that correspond to O─H and C═O moieties, respectively. Furthermore, the P 2p (134.2 eV, Figure 2e) and Si 2p (101.9 eV, Figure S11e) peaks are attributable to P─O and Si─O species. Taken collectively, these result confirm that the target hybrid structures had been successfully prepared [42].

The W 4f XPS spectrum of M‐PW_12_‐1D (Figure 2f; Table S5) shows two signals centered at 38.3 and 36.1 eV that correspond to the W 4f_5/2_ and W 4f_7/2_ states of W^VI^, while the two peaks at 37.8 and 35.6 eV are assigned to the W 4f_5/2_ and W 4f_7/2_ states of W^V^ [43]. M‐PW_12_‐1D exhibits W 4f peaks at higher binding energies than PW_12_, which is ascribable to strong electronic interactions and possible electron transfer between PW_12_ and the metal‐organic component, with electrons tending to migrate from the PW_12_ to the metal–organic component. M‐SiW_12_‐1D exhibited a trend similar to that observed for SiW_12_. The high‐resolution Ni 2p spectra of Ni‐PW_12_‐1D (Figure 2g) and Ni‐SiW_12_‐1D (Figure S11g) reveal characteristic Ni^2+^ species. Ni‐PW_12_‐1D exhibits Ni 2p_3/2_ and Ni 2p_1/2_ peaks at 855.5 and 873.5 eV, accompanied by satellite (sat.) features at 862.0 and 880.0 eV. Ni‐SiW_12_‐1D shows corresponding peaks at 856.2 (Ni 2p_3/2_) and 873.7 eV (Ni 2p_1/2_), with satellite peaks at 861.6 and 880.1 eV [44]. The Co 2p spectrum of Co‐PW_12_‐1D (Figure 2h) exhibits Co 2p_3/2_ and Co 2p_1/2_ peaks at 781.2 and 797.5 eV, along with satellite peaks at 786.8 and 803.6 eV, while Co‐SiW_12_‐1D (Figure S11h) exhibits main peaks at 781.6 (Co 2p_3/2_) and 797.4 eV (Co 2p_1/2_), with satellites at 786.7 and 803.5 eV, consistent with Co^2+^ species [45]. The deconvoluted Fe 2p spectra of Fe‐PW_12_‐1D (Figure 2i) and Fe‐SiW_12_‐1D (Figure S11i) show peaks at 710.3 (Fe 2p_3/2_) and 724.6 eV (Fe 2p_1/2_) for the former, and 709.5 (Fe 2p_3/2_) and 724.4 eV (Fe 2p_1/2_) for that latter, which are attributable to their corresponding Fe 2p orbitals. Meanwhile, the peaks observed at 714.6 (Fe‐PW_12_‐1D) and 716.3 eV (Fe‐SiW_12_‐1D) correspond to the satellite peaks of Fe^2+^ species [46]. Taken together, the XPS and FTIR results confirm that the metals and HNCP are coordinated.

### 2.2 Electrochemical Performance

**FIGURE 3:**
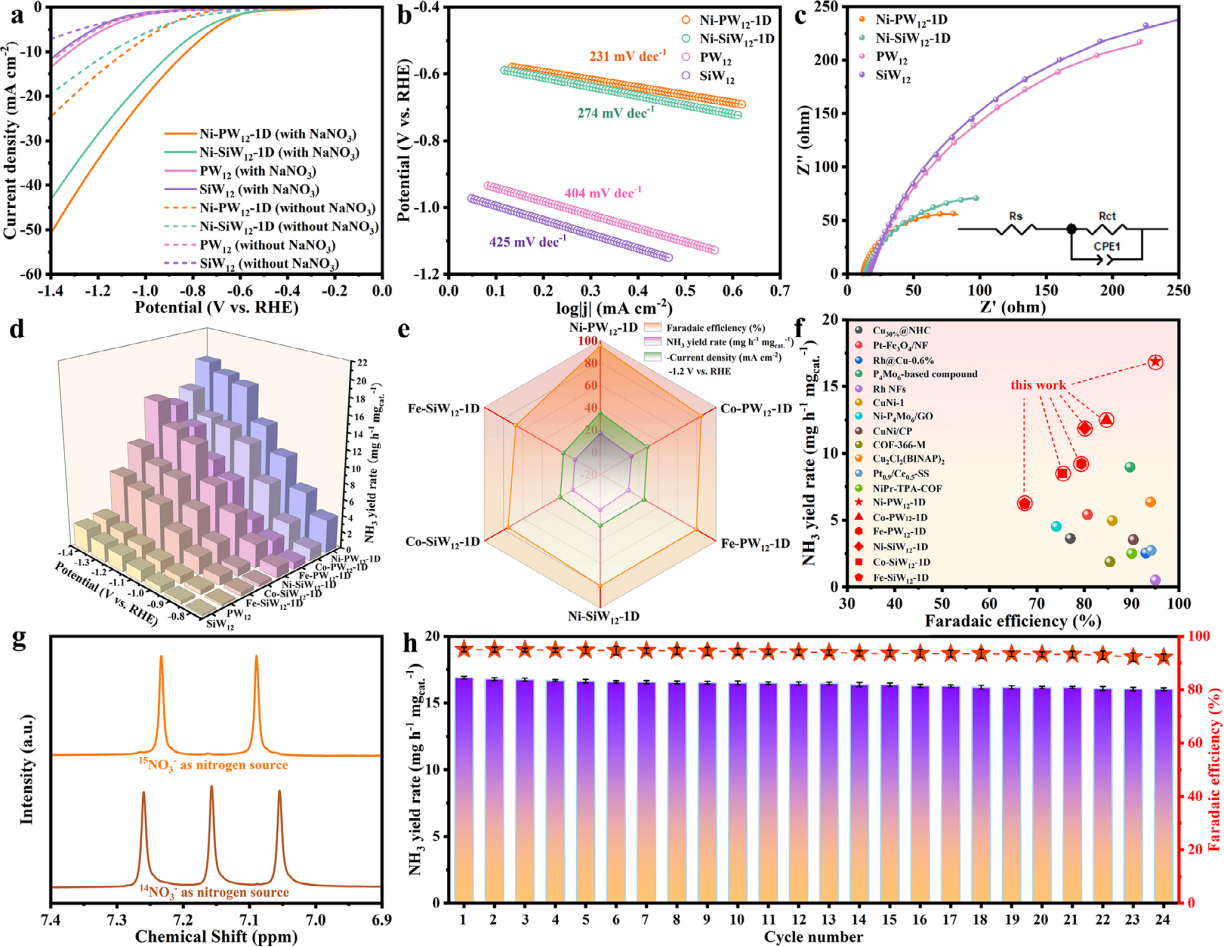
(a) LSV curves of PW_12_, SiW_12_, Ni‐PW_12_‐1D and Ni‐SiW_12_‐1D in 1 M Na_2_SO_4_ electrolyte with and without 1 M NaNO_3_. (b) Tafel plots and (c) EIS spectra in 0.1 M NaNO_3_/0.1 M Na_2_SO_4_. (d) NH_3_ yield rates of PW_12_, SiW_12_, M‐PW_12_‐1D and M‐SiW_12_‐1D. (e) Comparison of FE, NH_3_ yield and current density at −1.2 V vs. RHE over different catalysts. (f) Comparison of the ENRA performance of the catalysts in this work with that of previously reported catalysts in neutral media. (g) ^1^H NMR spectra of the electrolyte after ENRA at −1.2 V vs. RHE using ^15^NO^3–^ and ^14^NO^3–^ as the nitrate source. (h) Cyclic stability test on FE, and corresponding NH_3_ yield of ENRA at −1.2 V vs. RHE (three times error statistics).

The ENRA performance of PW_12_, SiW_12_, M‐PW_12_‐1D, and M‐SiW_12_‐1D was assessed using linear sweep voltammetry (LSV) in 0.1 M Na_2_SO_4_ and 0.1 m NaNO_3_/0.1 m Na_2_SO_4_ as electrolytes. LSV revealed a distinct enhancement in current density upon the introduction of NaNO_3_ into the electrolyte (Figure 3a; Figure S12), confirming high ENRA activity and low HER activity. Moreover, M‐PW_12_‐1D exhibited considerably higher current densities than M‐SiW_12_‐1D (Ni‐PW_12_‐1D > Co‐PW_12_‐1D > Ni‐SiW_12_‐1D > Fe‐PW_12_‐1D > Co‐SiW_12_‐1D > Fe‐SiW_12_‐1D > PW_12_> SiW_12_). ENRA Tafel slopes of 425, 404, 381, 367, 274, 287, 270, and 231 mV dec^−1^ were determined for SiW_12_, PW_12_, Fe‐SiW_12_‐1D, Co‐SiW_12_‐1D, Ni‐SiW_12_‐1D, Fe‐PW_12_‐1D, Co‐PW_12_‐1D, and Ni‐PW_12_‐1D, respectively (Figure 3b; Figure S13), revealing that Ni‐PW_12_‐1D exhibits the fastest ENRA reaction kinetics.

PW_12_, SiW_12_, M‐PW_12_‐1D, and M‐SiW_12_‐1D were subjected to electrochemical impedance spectroscopy with the aim of clarifying ENRA performance discrepancies, the Nyquist plots of which are shown in Figure 3c; Figure S14 along with an equivalent circuit comprising intrinsic resistance (R_s_) and charge‐transfer resistance (R_ct_) components. Notably, Ni‐PW_12_‐1D exhibited considerably lower R_s_ (11.9 Ω) and R_ct_ (129.7 Ω) values than Ni‐SiW_12_‐1D (13.4 and 183.4 Ω) and the other materials (Table S6), which implies that Ni‐PW_12_‐1D effectively enhances charge transport through the electrode and interface, thereby favoring ENRA [47].

ENRA experiments were conducted on the as‐prepared catalysts at applied potentials in the −0.8 to −1.4 V vs. RHE range, with the corresponding chronoamperometric (i‐t) curves recorded after 1 h of continuous electrolysis presented in Figure S15). NH_3_ and NO_2_^−^ concentrations were determined using colorimetric methods following electrolysis (Figures S16 and S17). All catalysts showed continuous rises in NH_3_ production with increasing applied potential, with maximum yields recorded at −1.4 V vs. RHE. Ni‐PW_12_‐1D delivered the highest NH_3_ yield of 18.6 mg h^−1^ mg_cat._^−1^, which is substantially higher than those of SiW_12_ (2.8 mg h^−1^ mg_cat._^−1^), PW_12_ (3.2 mg h^−1^ mg_cat._^−1^), Fe‐SiW_12_‐1D (7.7 mg h^−1^ mg_cat._^−1^), Co‐SiW_12_‐1D (10.5 mg h^−1^ mg_cat._^−1^), Ni‐SiW_12_‐1D (15.5 mg h^−1^ mg_cat._^−1^), Fe‐PW_12_‐1D (14.4 mg h^−1^ mg_cat._^−1^), and Co‐PW_12_‐1D (15.6 mg h^−1^ mg_cat._^−1^) (Figure 3d). Product selectivities were further examined by quantifying the NO_2_^−^ and H_2_ byproducts (Figure S18), which revealed that Ni‐PW_12_‐1D exhibited an NH_3_ FE that increased from 74.1% to 95.0% (Figure S18a). However, the NH_3_ FE was observed to decrease from 95.0% to 79.0% when the working potential was further increased from −1.2 V to −1.4 V vs. RHE, accompanied by a steady increase in H_2_ FE (from 2.0% to 14.5%), which is attributable to enhanced HER competitiveness. Ni‐PW_12_‐1D also exhibited the lowest NO_2_^−^ FE among the examined catalysts at the optimal potential of −1.2 V vs. RHE, which highlights its high selectivity toward ammonia. In contrast, the pristine POM exhibited a significantly higher H_2_ FE (up to 25.0%) at the same potential (−1.2 V vs. RHE), which indicates that the *H species on its surface preferentially participate in the HER process (Figure S18g). Notably, the *H intermediates are more effectively channeled into the ENRA pathway in Ni‐PW_12_‐1D, leading to effective HER suppression. Figure 3d and Figure S18 show that Ni‐PW_12_‐1D delivers a maximum NH_3_ yield rate of 16.9 mg h^−1^ mg_cat._^−1^ with an FE of 95.0% at −1.2 V vs. RHE, which are much higher than those of Ni‐SiW_12_‐1D (11.9 mg h^−1^ mg_cat._^−1^, 80.1%), Co‐SiW_12_‐1D (8.5 mg h^−1^ mg_cat._^−1^, 75.4%), Fe‐SiW_12_‐1D (6.2 mg h^−1^ mg_cat._^−1^, 67.4%), Co‐PW_12_‐1D (12.5 mg h^−1^ mg_cat._^−1^, 84.7%), Fe‐PW_12_‐1D (9.2 mg h^−1^ mg_cat._^−1^, 79.4%), PW_12_ (2.7 mg h h^−1^ mg_cat._^−1^, 51.0%), and SiW_12_ (2.1 mg h^−1^ mg_cat._^−1^, 50.7%). These results show that the target trinuclear POMOC catalyst is more selective and specific toward ENRA compared to the pure POMs and other dinuclear POMOCs, highlighting the essential role played by tailored metal centers in promoting ENRA over the HER. Ni‐PW_12_‐1D exhibited outstanding catalytic performance at −1.2 V vs. RHE, as summarized in Figure 3e, including an NH_3_ FE of 95.0%, a yield of 16.9 mg h^−1^ mg_cat._^−1^, and a current density of −35.1 mA cm^−2^, which surpasses the performance of M‐SiW_12_‐1D, Co‐PW_12_‐1D, and Fe‐PW_12_‐1D. More notably, Figure 3f and Table S7 reveal that Ni‐PW_12_‐1D surpasses the performance of most reported ENRA electrocatalysts in neutral media, which underscores its state‐of‐the‐art characteristics.

To further highlight the practical relevance of Ni‐PW_12_‐1D for real wastewater treatment, the ENRA performance under varying nitrate concentrations (10–100 mM, Figure S19) warrants in‐depth connection to actual wastewater characteristics. Most domestic wastewater and low‐strength industrial effluents typically contain nitrate at 10–50 mM, far lower than the 100 mM often used in laboratory evaluations. Notably, even at 10 mM, Ni‐PW_12_‐1D still achieves an NH_3_ yield rate of 4.2 mg h^–^^1^ mg^cat.–^ ^1^ and a FE of 62.9%. This performance is critical for practical deployment, as it eliminates the need for energy‐intensive nitrate pre‐concentration steps that are mandatory for catalysts with poor low‐concentration activity, thereby reducing both capital and operational costs of wastewater treatment systems.

We determined the NH_3_ nitrogen using ^15^N isotope tracing experiments with Na^15^NO_3_ instead of Na^14^NO_3_ during ENRA, with ^1^H NMR spectroscopy used to detect the produced NH_3_. The ^1^H NMR spectrum of the electrolyte exhibited characteristic triplets and doublets that corresponding to ^15^NH_4_^+^ and ^14^NH_4_^+^, respectively (Figure 3g) [48, 49], which confirms that the NH_3_ originates exclusively from the electroreduction of NO_3_^−^ rather than contamination. Figure S20) displays NH_3_ yields under various test conditions: 0.1 m Na_2_SO_4_ (with catalyst), 0.1 m NaNO_3_/0.1 m Na_2_SO_4_ (with catalyst), and 0.1 m NaNO_3_/0.1 m Na_2_SO_4_ (without catalyst). The control group delivered negligible NH_3_ yields relative to those observed for 0.1 m NaNO_3_/0.1 m Na_2_SO_4_ (with catalyst), thereby confirming that the NH_3_ produced in the electrolyte arises from the reduction of nitrate catalyzed by Ni‐PW_12_‐1D.

Furthermore, the stability of Ni‐PW_12_‐1D toward ENRA was assessed via successively cycling the catalyst 24‐times at −1.2 V vs. RHE. Figure 3h shows that both the FE and NH_3_ yield rates were only slightly different after 24 cycles compared to the initial values, which indicates that Ni‐PW_12_‐1D is highly electrocatalytically stable toward ENRA. Notably, Ni‐PW_12_‐1D exhibited an i‐t curve that displays a relatively stable current density even after 24 h of continuous testing at −1.2 V vs. RHE (Figure S21a), highlighting the remarkable long‐term stability of this catalyst. Ni‐PW_12_‐1D was examined by XRD and FTIR spectroscopy, which revealed negligible structural differences between the catalyst before and after ENRA (Figure S21b: Figure S21c). Finally, XPS revealed minimal variations in elemental composition and valence states (Figure S22), further confirming the compositional stability of Ni‐PW_12_‐1D under ENRA conditions.

### 2.3 Electrocatalytic Reduction Mechanism

**FIGURE 4:**
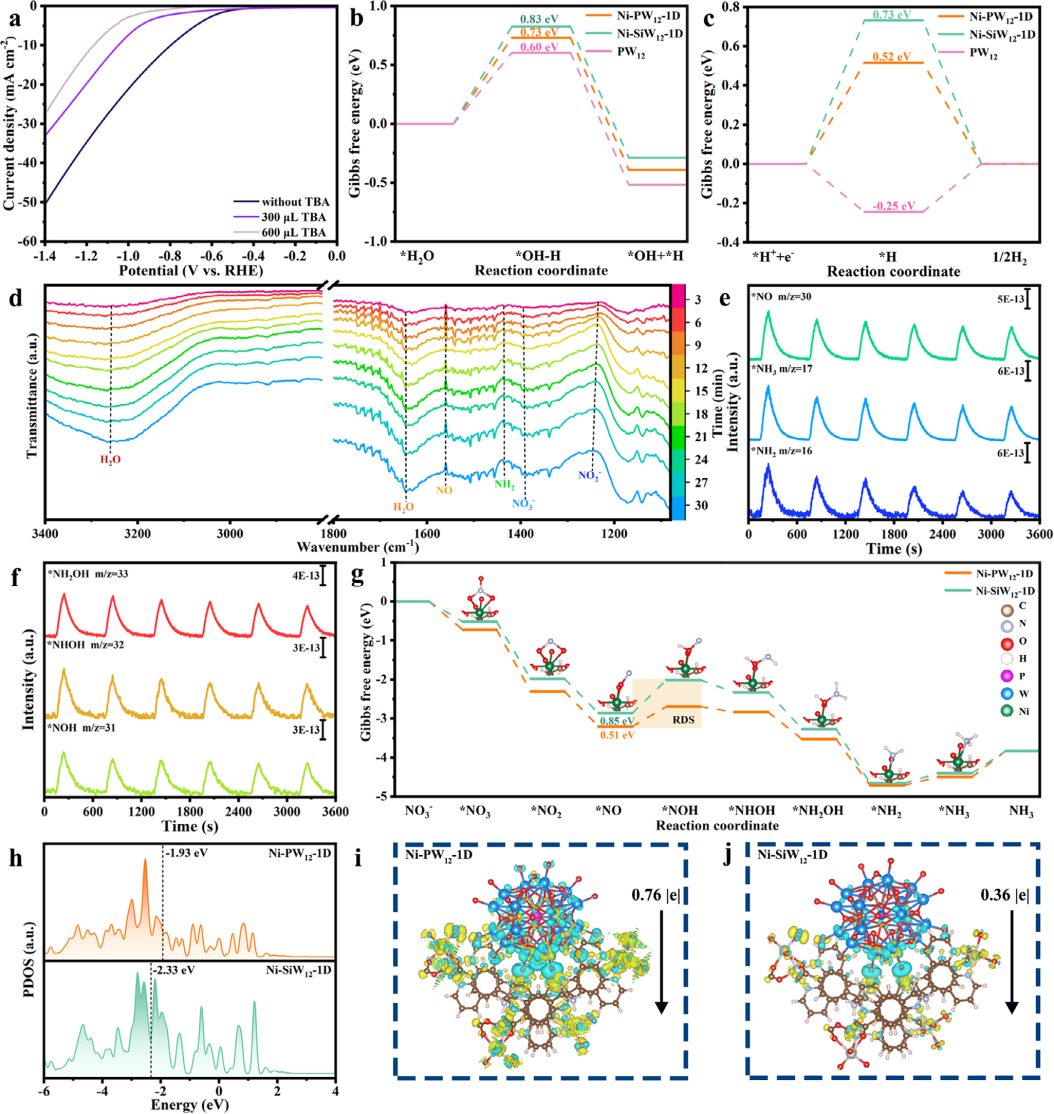
(a) LSV plots of M‐PW_12_ with 300 µL, 600 µL and without TBA. (b) The calculated ΔG profiles in water‐splitting reaction on Ni‐PW_12_‐1D and Ni‐SiW_12_‐1D, respectively. (c) The calculated ΔG profiles in hydrogen evolution reaction on Ni‐PW_12_‐1D and Ni‐SiW_12_‐1D, respectively. (d) Electrochemical in situ FTIR spectra of ENRA on Ni‐PW_12_‐1D at −1.2 V vs. RHE with different electrolysis durations. (e, f) Electrochemical online DEMS results for the ENRA over Ni‐PW_12_‐1D. (g) Gibbs free energies of ENRA for different intermediates on Ni‐PW_12_‐1D and Ni‐SiW_12_‐1D, respectively, and the structures of ENRA intermediates adsorbed on Ni‐PW_12_‐1D. (h) Projected density of states of Ni‐PW_12_‐1D and Ni‐SiW_12_‐1D, respectively. Charge density differences and charge transfer of (i) Ni‐PW_12_‐1D and j) Ni‐SiW_12_‐1D. Yellow and cyan colors represent charge accumulation and depletion, respectively.

Previous studies showed that two main processes are involved in ENRA under neutral conditions: deoxidation and hydrogenation, in which *H plays an important role. First clarifying related reaction mechanisms is important in order to further investigate the mechanism by which the Ni‐PW_12_‐1D catalyst produces *H in neutral media. *H generation during the nitrate reduction reaction in neutral media has previously been rationalized as a semi‐HER process. Specifically, the catalyst first undergoes a Volmer reaction (H_2_O + e^−^ → *H_ads_ + OH^−^) that splits water and generates *H, which is adsorbed onto the catalyst surface where it combines with deoxygenated intermediates in the hydrogenation process. However, the lower degree of ionization in neutral media limits the charge‐transfer rate, which slows the water‐splitting kinetics that supplies protons, leading to only a small amount of *H participating in the reaction. LSV traces were acquired for Ni‐PW_12_‐1D under ENRA conditions in the presence and absence of *tert*‐butanol (TBA), a commonly used quenching agent for hydrogen radicals. Figure 4a shows that current density produced by Ni‐PW_12_‐1D gradually declines when 300 and 600 µL of TBA are added; hence ENRA is significantly inhibited owing to the lower availability of active hydrogen. These results highlight the essential role played by *H in the nitrate‐to‐ammonia conversion pathway.

We subjected the (001) crystal facets of Ni‐PW_12_‐1D and Ni‐SiW_12_‐1D to density functional theory (DFT) calculations with the aim of establishing a computationally reliable model that is aligned with experimental reality; this choice is supported by prior PXRD characterization data that confirm that the (001) facet is the predominantly exposed plane of the as‐synthesized catalysts, thereby ensuring that the model reflects the actual surface structures of the experimental samples.

Subsequent DFT calculations further probed the water splitting and HER processes over PW_12_, Ni‐PW_12_‐1D, and Ni‐SiW_12_‐1D. Figure 4b shows that the PW_12_ surface exhibited an energy barrier for water splitting of only 0.60 eV, which is lower than those of Ni‐PW_12_‐1D (0.73 eV) and Ni‐SiW_12_‐1D (0.83 eV). Consequently, PW_12_ is expected to efficiently facilitate the water‐splitting process. Notably, Ni‐SiW_12_‐1D exhibited the least‐favorable water‐splitting capability among the three samples; nevertheless, the water‐splitting capability of Ni‐PW_12_‐1D is significantly superior than that of Ni‐SiW_12_‐1D, and is ascribable to the incorporation of PW_12_, which remarkably lowers the energy barrier for water splitting. Figures S23 and S24) show side views depicting intermediate adsorption on the Ni‐PW_12_‐1D surface that reveal that water molecules are preferentially adsorbed onto the PW_12_ component of the catalyst. The *H species migrate to the surface of Ni‐PW_12_‐1D to support subsequent ENRA processes after the water molecules are cleaved by PW_12_ into rich *H and OH^−^ species. The Volmer step over PW_12_ is exothermic (Figure 4c), with an energy barrier of −0.25 eV, which facilitates *H generation. In contrast, Ni‐PW_12_‐1D and Ni‐SiW_12_‐1D exhibit endothermic Volmer steps; specifically, Ni‐PW_12_‐1D is associated with a lower energy barrier (0.52 eV) than Ni‐SiW_12_‐1D (0.73 eV), which implies that *H is more likely to be generated over the former. The Heyrovsky step (*H_ads_ + H⁺ + e^−^ → H_2_) on Ni‐PW_12_‐1D is calculated to have a higher energy barrier (−0.52 eV) than on Ni‐SiW_12_‐1D (−0.73 eV) [50], which demonstrates that Ni‐PW_12_‐1D not only better facilitates the formation of *H intermediates than Ni‐SiW_12_‐1D, but also suppresses the competing HER.

To clarify the origin of abundant active *H on Ni‐PW_12_‐1D, contact angle (CA) tests were conducted to evaluate the hydrophilicity of PW_12_ and Ni‐PW_12_‐1D. Hydrophilicity correlates closely with water adsorption and dissociation, which is the primary *H source for ENRA. As shown in Figure S25, PW_12_ exhibits pronounced hydrophilicity with a contact angle (CA) of 18°at 1 s, a property that correlates with its robust intrinsic *H generation capability. Yet the generated *H species readily recombine to form H_2_, leading to low utilization efficiency of the active *H intermediates. In contrast, Ni‐PW_12_‐1D displays moderate hydrophilicity (CA = 23° at 1 s); this modulation of hydrophilicity, combined with the incorporation of integrated Ni‐based metal‐organic clusters, ensures adequate water adsorption (a prerequisite for *H generation) while inhibiting the undesired conversion of *H to H_2_. This experimental observation is consistent with DFT calculations regarding the optimized water splitting process and *H migration behavior.

A series of in situ FTIR spectra were acquired to elucidate the ENRA pathway associated with Ni‐PW_12_‐1D (Figure 4d). The absorption band observed at 1387 cm^−1^ is assigned to nitrate ions (NO_3_^−^). At the same time, several other absorption peaks that correspond to reduction intermediates (NO* at 1560 cm^−1^ and NO_2_^−^ at 1239 cm^–^^1^) [51, 52], hydrogenation intermediates (NH_2_* at 1439 cm^−1^), and adsorbed water (at 1644 and 3255 cm^−1^) [53] were observed and whose intensities gradually increased with reaction time.

Differential electrochemical mass spectrometry (DEMS) was used to detect signals that correspond to the reaction intermediates formed during ENRA (Figures 4e,f). Signals at m/z values of 33, 32, 31, 30, 17, and 16 correspond to *NH_2_OH, *NHOH, *NOH, *NO, *NH_3_, and *NH_2_, respectively; these signals were observed over six consecutive cycles. Furthermore, *NO is first hydrogenated to *NOH under the in situ operating conditions, followed by further hydrogenation to form *NHOH and *NH_2_OH, the latter of which then undergoes deoxygenation to form *NH, which is then hydrogenated to NH_3_. To rule out interference from background noise or side reactions, a control experiment (DEMS analysis of the electrolyte in the absence of nitrate) was conducted, and no characteristic m/z signals were detected (Figure S26). This directly confirms that all detected signals in the original tests originate exclusively from the electrocatalytic nitrate reduction reaction, rather than background noise. Based on these observations, we proposed the following reaction pathway: NO_3_^−^ → *NO_3_ → *NO_2_ → *NO → *NOH→ *NHOH → *NH_2_OH → *NH_3_ → NH_3_.

DFT calculations were performed to gain an in‐depth understanding of the enhanced ENRA activity of Ni‐PW_12_‐1D, with Gibbs free energy (ΔG) profiles obtained for Ni‐PW_12_‐1D and Ni‐SiW_12_‐1D along the ENRA pathway. All reaction steps, from *NO_3_^−^ to *NH_3_, are described in detail in these free energy diagrams (Figure 4 g; Figure S27). Notably, the *NO → *NOH step is the rate‐determining step (RDS) for both catalysts, and corresponds to the first hydrogenation step in the entire reaction sequence. The RDS over Ni‐PW_12_‐1D was calculated to have a ΔG of only 0.51 eV, which is significantly lower than that of Ni‐SiW_12_‐1D (0.85 eV). These distinctly different RDS barriers are mainly responsible for the superior ENRA performance of Ni‐PW_12_‐1D compared to that of Ni‐SiW_12_‐1D.

To examine the impact of POMs on the electronic structure of POMOCs, we analyzed and compared the projected density of states (PDOS) of Ni‐PW_12_‐1D, Co‐PW_12_‐1D, Fe‐PW_12_‐1D, and Ni‐SiW_12_‐1D. As illustrated in Figure 4 h and Figure S28), the d‐band centers of these materials are −1.93 eV (Ni‐PW_12_‐1D), −1.94 eV (Co‐PW_12_‐1D), −2.00 eV (Fe‐PW_12_‐1D), and −2.33 eV (Ni‐SiW_12_‐1D), respectively. This sequence (Ni‐PW_12_‐1D > Co‐PW_12_‐1D > Fe‐PW_12_‐1D) in d‐band center proximity to the Fermi level is perfectly consistent with the ENRA performance trend observed in our experiments. Incorporation of PW_12_ induces an upward shift of the d‐band in the 3d orbitals of Ni, Co, and Fe, with the d‐band center of Ni‐PW_12_‐1D shifted closest to the Fermi level, followed by Co‐PW_12_‐1D and Fe‐PW_12_‐1D, while Ni‐SiW_12_‐1D exhibits the most downward‐shifted d‐band center. This results in reduced occupancy of antibonding orbitals for the PW_12_‐based POMOCs, promoting bond formation with adsorbed intermediates and thereby enhancing the adsorption capacity for intermediates in ENRA [54]. In addition, we also used charge density difference calculations to investigate the electronic structures of Ni‐PW_12_‐1D and Ni‐SiW_12_‐1D and elucidate the underlying mechanism, with regions of charge accumulation shown in yellow and charge‐depleted ones shown in cyan (Figures 4i,j). Notably, the POM units donate electrons to the MOC in both materials, consistent with the XPS results [55]. The POM in the Ni‐PW_12_‐1D structure transfers more electrons (0.76 e^−^) to the MOC than the POM in Ni‐SiW_12_‐1D according to Bader‐charge‐analysis data. Such differences in electron‐transfer efficiency are attributable to variations in the electronic structures of PW_12_ and SiW_12_.

## 3 Conclusion

We rationally designed a series of dinuclear and trinuclear chain‐like POMOCs with the aim of catalyzing ENRA in neutral media. Notably, the trinuclear Ni‐PW_12_‐1D complex exhibited excellent ENRA performance, delivering a significantly high NH_3_ FE of 95.0% and an NH_3_ yield of 16.9 mg h^−1^ mg_cat._^−1^ at −1.2 V vs. RHE. A combination of experimental probes, in situ FTIR spectroscopy, DEMS, and DFT calculations revealed that synergy between the POM unit and metal–organic complex not only facilitates proton‐coupled electron transfer that generates active *H, but also optimizes the balance between hydrogenation and the competitive HER. Moreover, electron transfer from the POM to the MOC active sites effectively modulates the local electron density, thereby lowering the energy barrier of the rate‐determining step (*NO → *NOH) and promoting the overall ENRA pathway. These findings elucidate the critical function of the multinuclear POMOC architecture in enabling efficient nitrate‐to‐ammonia conversion and provide a strategic direction for the designs of advanced multicomponent electrocatalysts for sustainable nitrogen cycling.

## 4 Experimental Section

See Supporting Information for details.

## Conflicts of Interest

The authors declare no conflicts of interest.

## Supporting information




**Supporting File 1**: advs73540‐sup‐0001‐SuppMat.docx.

**Supporting File 2**: advs73540‐sup‐0002‐1‐3.cif.

## Data Availability

The data that support the findings of this study are available from the corresponding author upon reasonable request.
