# Supplementary material for: Polyoxometalate as Hydrogen–Electron Hub in Metal–Organic Complex for Electrocatalytic Nitrate Reduction to Ammonia
Source: Adv Sci (Weinh). 2025 Dec 22;13(14):e20543. doi: 10.1002/advs.202520543 (PMC12970284; doi:10.1002/advs.202520543)
Supplement: Supplementary file 1 — Supporting File 1: advs73540‐sup‐0001‐SuppMat.docx. [file ADVS-13-e20543-s002.docx]

Supporting Information

Polyoxometalate as Hydrogen-Electron Hub in Metal-Organic Complex for Electrocatalytic Nitrate Reduction to Ammonia

Qiushuang Jiang, Xinming Wang*, Shengji Tian, Haijun Pang, Yongbin Song, and Huiyuan Ma

Q. Jiang, X. Wang, H. Pang, Y. Song, H. Ma

School of Materials Science and Chemical Engineering, Harbin University of Science and Technology, Harbin 150040, P. R. China

E-mail: wangxinming20@126.com

S. Tian

School of Chemistry and Chemical Engineering, Harbin Institute of Technology, Harbin, 150001 P. R. China

**Experimental section**

**1. Materials**

Nafion per-fluorinated resin solution, nickel chloride hexahydrate (NiCl_2_·6H_2_O, AR), cobalt chloride hexahydrate (CoCl_2_·6H_2_O, AR), Ferric chloride hexahydrate (FeCl_3_·6H_2_O, AR), 2-(4-carboxyphenyl)imidazo(4,5-f)(1,10) phenanthroline (C_20_H_12_N_4_O_2_, AR), phosphotungstic acid hydrate (H_3_O_40_PW_12_·xH_2_O, AR), tungstosilicic acid hydrate (H_4_O_40_SiW_12_·xH_2_O, AR), sodium pentacyanonitrosylferrate(III) dihydrate (Na_2_[Fe(CN)_5_NO]·2H_2_O), N-(1-naphthyl) ethylenediamine dihydrochloride(C_12_H_14_N_2_·2HCl, AR), dimethyl sulfoxide-d6 (DMSO-*d*_6_, deuterium for 99.9 %), tertiary butanol(C_4_H_10_O, AR), ethanol (C_2_H_5_OH, AR) and isopropyl alcohol (C_3_H_8_O, AR) were obtained from Tianjin Fuyu Fine Chemical Co., China. Sodium nitrate (NaNO_3_, AR), sodium nitrite (NaNO_2_, AR), sodium hypochlorite (NaClO, AR), sulfanilic acid (C_6_H_7_NO_3_S, AR), salicylic acid (C_7_H_6_O_3_, AR), and potassium bromide (KBr, AR) were obtained from Shanghai McLean Co., China. Sodium sulphate (Na_2_SO_4_, AR) and phosphoric acid (H_3_PO_4_, AR) were purchased from Tianjin Guangfu Technology Development Co., China. Sodium citrate dihydrate (C_6_H_5_Na_3_O_7_∙2H_2_O, AR) was obtained from Sigma-Aldrich, USA. Sodium hydroxide (NaOH, AR) was obtained from Shanghai Yien Chemical Technology Co., China. Argon (Ar, high-purity, 99.9999 %) was purchased from Qing Hua Gas Co., China. Sodium nitrate-^15^N (Na^15^NO_3_, AR) and ammonium chloride (NH_4_Cl, AR) were purchased from Aladdin. All samples were used without further purification. The deionized water was produced using an ultrapure water system.

**2. Experimental section**

**2.1. Synthesis of [Ni_1.5_(H_2_O)_2_C_20_O_3_N_4_H_11_]_4_[PW^VI^_7_W^V^_5_O_40_]**

A mixture of H_3_O_40_PW_12_·xH_2_O (200 mg, 0.07 mmol), HNCP = 4-(1H-imidazo[4,5-f][1,10]phenanthrolin-2-yl) benzoic acid (40 mg, 0.12 mmol), NiCl_2_·6H_2_O (475.4 mg, 2 mmol), 10 mL of C_2_H_5_OH, and 40 mL of deionized water was vigorously stirred for 1 h, then adjusted the pH of solution to 2.4 to 3.2 with 1.0 M H_3_PO_4_ or NaOH. Subsequently, the suspension was transferred to a 100 mL Teflon-lined autoclave, where it was maintained at 180 °C for 5 days. After slow cooling to room temperature, green cuboid crystals of Ni-PW_12_-1D were obtained (51% yield based on PW_12_, washed with distilled water, and dried at room temperature. Anal. calcd. for C_80_H_60_N_16_O_60_PW_12_Ni_6_: C, 20.04; N, 4.67; O, 20.02; P, 0.65; W, 46.01; Ni, 7.35. Found (%): C, 20.16; N, 4.34; O, 20.18; P, 0.77; W, 46.27; Ni, 7.12.

**2.2. Synthesis of [Co_1.5_(H_2_O)_2_C_20_O_3_N_4_H_11_]_4_[PW^VI^_7_W^V^_5_O_40_]**

The preparation of Co-PW_12_-1D was like the method of Ni-PW_12_-1D. Instead of NiCl_2_·6H_2_O, CoCl_2_·6H_2_O (475.9 mg, 2 mmol) as a substitute was added in solution. Finally, orange cuboid crystals were obtained (46% yield based on PW_12_)

**2.3. Synthesis of [Fe_1.5_(H_2_O)_2_C_20_O_3_N_4_H_11_]_4_[PW^VI^_7_W^V^_5_O_40_]**

The preparation of Fe-PW_12_-1D was like the method of Ni-PW_12_-1D. Instead of NiCl_2_·6H_2_O, FeCl_3_·6H_2_O (540.6 mg, 2 mmol) as a substitute was added in solution. Finally, red cuboid crystals were obtained (53% yield based on PW_12_)

**2.4. Synthesis of [Ni(H_2_O)_2_C_20_O_2_N_4_H_11_]_4_[SiW^VI^_12_O_40_]**

The preparation of Ni-SiW_12_-1D was like the method of Ni-PW_12_-1D. Instead of H_3_O_40_PW_12_·xH_2_O, H_4_O_40_SiW_12_·xH_2_O (200 mg, 0.07 mmol) as a substitute was added in solution. Finally, green cuboid crystals were obtained (48% yield based on SiW_12_). Anal. calcd. for C_80_H_60_N_16_O_56_SiW_12_Ni_4_: C, 20.84; N, 4.86; O, 19.43; Si, 0.61; W, 47.85; Ni, 5.09. Found (%): C, 20.63; N, 4.94; O, 19.31; Si, 0.75; W, 47.96; Ni, 5.21.

**2.5. Synthesis of [Co(H_2_O)_2_C_20_O_2_N_4_H_11_]_4_[SiW^VI^_12_O_40_]**

The preparation of Co-SiW_12_-1D was like the method of Ni-SiW_12_-1D. Instead of NiCl_2_·6H_2_O, CoCl_2_·6H_2_O (475.9 mg, 2 mmol) as a substitute was added in solution. Finally, orange cuboid crystals were obtained (61% yield based on SiW_12_). Anal. calcd. for C_80_H_60_N_16_O_56_SiW_12_Co_4_: C, 20.84; N, 4.86; O, 19.43; Si, 0.61; W, 47.84; Co, 5.11. Found (%): C, 20.63; N, 4.73; O, 19.28; Si, 0.58; W, 47.70; Co, 5.28.

**2.6. Synthesis of [Fe(H_2_O)_2_C_20_O_2_N_4_H_11_]_4_[SiW^VI^_12_O_40_]**

The preparation of Fe-SiW_12_-1D was like the method of Ni-SiW_12_-1D. Instead of NiCl_2_·6H_2_O, FeCl_3_·6H_2_O (540.6 mg, 2 mmol) as a substitute was added in solution. Finally, red cuboid crystals were obtained (42% yield based on SiW_12_)

**3. Characterization**

Single-crystal XRD data were measured on a Bruker APEXII CCD diffractometer with graphite-monochromated Mo Kα radiation (λ = 0.71073 Å) at 296 K. Powder X-ray diffraction (PXRD) was performed using a Rigaku D/Max-cA X-Ray diffractometer with Ni-filtered Cu Kα radiation in the 2θ range of 5-50°. The X-ray photoelectron spectroscopy (XPS) was carried out on an instrument of ESCALAB 250Xi with Al Kα as the excitation source. The binding energies were calibrated with C1s peak positioned at 284.8 eV. Scanning electron microscope (SEM) measurements were performed on a Regulus8100 cold filed emission scanning electron microscope. The TENSOR II Fourier transform infrared (FTIR) spectrometer was used for functional groups analysis of the photocatalysts. Ultraviolet–visible (UV-Vis) spectrophotometer (SolidSpec-3700) was utilized to obtain the optical absorption characteristics within 200-800 nm. ^1^H nuclear magnetic resonance (NMR) experiments were carried out at 303 K for 5 % w/v sample solution in DMSO-*d*6 using Bruker Avance NEO 300. The gaseous products for electrocatalytic reaction were quantified on a GC-9800 gas chromatograph.

**4. Electrochemical measurements**

The electrochemical measurements were conducted by a CHI 760 electrochemical analyzer using a typical three-electrode H-type electrochemical. For the ENRA three-electrode system, the catalyst-modified carbon cloth electrode (denoted as CCE, 1 cm×1 cm), Ag/AgCl electrode and Pt foil were used as working, reference and counter electrodes, respectively. The potentials reported in this work were converted to RHE scale via calibration with the following equation: E (νs. RHE) = E (νs. Ag/AgCl) + 0.197 + 0.059×pH. Before all measurements, the cathode chamber was purged with Ar (99.99 %) gas to purify the electrolyte for approximately 30 min. Linear scanning voltammetry (LSV) was repeated at a rate of 5 mV s^−1^ until the curves basically coincide. Chronoamperometry (i-t) measurement was carried out at different applied potentials for 1 h. Electrochemical impedance spectroscopy (EIS) measurements were carried out from 0.1 Hz to 1000 kHz with an amplitude of 10 mV at the open-circuit voltage. The electrochemically active surface area (ECSA) was estimated by measuring the double-layer capacitance (C_dl_) via the CV test at the scan rates of 10-100 mV s^–1^. The following well-known equation was used to calculate the Tafel slope of the catalysts. η=b log |j| + a. where “b” is the Tafel slope, “j” is the current density, “η” is the near zero onset overpotential, and “a” is a constant.

**5. Preparation of the Modified Electrodes**

Given the poor intrinsic electrical conductivity of POM-based metal–organic complexes (POMOCs) were introduced to mix with the as-synthesized POMOCs to improve the conductivity. Nafion solution was introduced as a kind of POMOCs dispersion solution generally applied in many reported works, which can form a homogeneous ink with POMOCs and further help to attach onto the surface of carbon cloth. The preparation of the CCE working electrode was as follows. A volume of 3 mg of electrocatalyst, 125 µL of isopropyl alcohol, 365 µL of DI and 10 µL of Nafion solution (0.1 wt% water solution) are grounded to form uniform catalyst ink. After sonication for 2 h, 50μL of ink was dropped directly onto both sides of a carbon cloth (1 cm × 1 cm) with a catalyst loading density of ~0.3 mg·cm^–2^ and dried.

**6. Quantification of ion concentration**

**6.1. Quantification of NH_3_**

The production of ammonia in the electrolyte was measured using, a modified indophenol blue method. 2 mL electrolyte was removed from the cathodic chamber and mixed with oxidizing solution containing 2 mL of NaOH solution (1.0 M) containing C_7_H_6_O_3_ (5 wt %), C_6_H_5_NaO_7_∙2H_2_O (5 wt %), 1 mL of NaClO (0.05 M), and 0.2 mL of 1 wt % Na_2_[Fe(CN)_5_NO]·2H_2_O for 2 h. The solution was measured with UV–vis absorption spectrum at 655 nm. Calibration curve of NH_3_ in electrolyte solution was plotted using a series of different concentration of standard NH_4_Cl solution diluted by 0.1 M Na_2_SO_4_. The linear relationship is y = 0.487x+0.090, R^2^ = 0.997 in neutral electrolyte.

**6.2. Quantification of NO_2_^–^**

The produced NO_2_^–^ was carried out by the Griess method. A mixed solution was fabricated by adding 0.05 g C_12_H_14_N_2_·2HCl, 5 g C_6_H_7_NO_3_S and 50 mL C_7_H_6_O_3_ to a volumetric flask and diluting to 1 L with a certain amount of DI. Then, the mixed solution and DI were mixed in a volume ratio of 4:1 to obtain the color reagent. 9 mL of color reagent was added to 1 mL of standard solution and aged for 15 min. The solution was measured with UV–vis absorption spectrum at 540 nm. Calibration curve of NaNO_2_ in 0.1 M Na_2_SO_4_ was plotted using a series of different concentration of standard NaNO_2_ stock solution diluted by 0.1 M Na_2_SO_4_. The fitting curve shows good linear relation of absorbance with NaNO_2_ concentration. The linear relationship is y = 0.422x+0.082, R^2^=0.998 in neutral electrolyte.

**6.3. Determination of FE and NH_3_ yield rate**

The calculation equations of electrochemical nitrate reduction reaction to ammonia (ENRA) are as follows:

Faradaic efficiency ($\text{FE}_{\text{NH}_{\text{3}}}$) = 8 F × ($\text{C}_{\text{NH}_{\text{3}}}$× V)/($\text{M}_{\text{NH}_{\text{3}}}$× Q)

Ammonia yield rate ($\text{Yield}_{\text{NH}_{\text{3}}}$) = ($\text{C}_{\text{NH}_{\text{3}}}$× V)/(t × m_cat._)

where F is the Faraday constant (96485 C·mol^–1^), Q is the charge accumulated *via* 1 h electrocatalysis, and V is the volume of the Na_2_SO_4_ electrolyte (50 mL), $\text{C}_{\text{NH}_{\text{3}}}$ is the measured NH_3_ concentration, $\text{M}_{\text{NH}_{\text{3}}}$ is the molar mass of NH_3_ (17 g·mol^–1^), t is the reduction reaction time (1 h) and m_cat._ is the loaded quality of catalyst.

Faradaic efficiency ($\text{FE}_{\text{NO}_{\text{2}}^{-}}$) = 2 F × ($\text{C}_{\text{NO}_{\text{2}}^{-}}$× V)/($\text{M}_{\text{NO}_{\text{2}}^{-}}$× Q)

Ammonia yield rate ($\text{Yield}_{\text{NO}_{\text{2}}^{-}}$) = ($\text{C}_{\text{NO}_{\text{2}}^{-}}$× V)/(t × m_cat._)

where $\text{C}_{\text{NO}_{\text{2}}^{-}}$is the measured NO_2_^–^ concentration, $\text{M}_{\text{NO}_{\text{2}}^{-}}$is the molar mass of NO_2_^–^ (46 g·mol^–1^).

**6.4. Determination of hydrogen**

Firstly, 300 μL gas was taken out from the electrolytic cell and injected into the gas chromatography. Then the peak areas of H_2_ were recorded. The production of the hydrogen curve was calibrated using a series of standard volume H_2_ of 0 mL, 0.1 mL, 0.2 mL, 0.3 mL, and 0.4 mL, respectively.

Faradaic efficiency ($\text{FE}_{\text{H}_{\text{2}}}$) = 2 F × $\text{C}_{\text{H}_{\text{2}}}$/(22.4 × Q)

Where $\text{C}_{\text{H}_{\text{2}}}$ is the volume of H_2_ (L).

**6.5. Determination of other products**

The faradaic efficiency of other products (e.g. N_2_, N_2_H_4_, NO_2_ etc.) was the remaining percentage apart from $\text{FE}_{\text{NH}_{\text{3}}}$, $\text{FE}_{\text{NO}_{\text{2}}^{-}}$ and $\text{FE}_{\text{H}_{\text{2}}}$ based on the equation:

Faradaic efficiency (FE_others_) = 100% - $\text{FE}_{\text{NH}_{\text{3}}}$ - $\text{FE}_{\text{NO}_{\text{2}}^{-}}$ - $\text{FE}_{\text{H}_{\text{2}}}$

**7. Isotope Labeling Experiments**

The isotope tracing experiments were all conducted at reduce potential -1.2 V (vs. RHE) for 4h. The catholyte was 0.1 M Na^15^NO_3_ (^15^N ≥ 99% atom) + 0.1M Na_2_SO_4_. After electrocatalysis, a certain amount catholyte was taken out for the NMR detection.

**8. In situ electrochemical characterization**

**8.1 Online differential electrochemical mass spectroscopy measurement**

Differential electrochemical mass spectroscopy (DEMS) measurement was performed on a homemade electrochemical cell equipped with a peristaltic pump. Catalyst-coated carbon paper, Pt wire, and Ag/AgCl electrode were used as the working, counter electrode, and reference electrode, respectively. The potentiostatic measurement was adopted. After the baseline remained stable, the potential switch was turned on and off periodically. Six cycles of mass signals were collected to avoid accidental errors during the DEMS measurements.

**8.2 In situ** **Fourier transform infrared**

Electrochemical in situ Fourier transform infrared (FTIR) spectroscopy was carried out on a Nicolet Nexus 670 spectrometer using a mercury-cadmium-telluride (MCT) detector cooled by liquid nitrogen. Catalyst-coated Au-modified single-bounce silicon, Pt wire, and SCE were used as the working, counter, and reference electrodes, respectively. The potentiostatic measurement technology was adopted.

**9. Theoretical calculations**

The Vienna Ab Initio Package (VASP) was employed to perform all the density functional theory (DFT) calculations within the generalized gradient approximation (GGA) using the Perdew, Burke, and Enzerhof (PBE) formulation.^[1-3]^ The projected augmented wave (PAW) potentials were applied to describe the ionic cores and take valence electrons into account using a plane wave basis set with a kinetic energy cutoff of 450 eV.^[4,5]^ Partial occupancies of the Kohn-Sham orbitals were allowed using the Gaussian smearing method and a width of 0.05 eV. The electronic energy was considered self-consistent when the energy change was smaller than 10^–5^ eV. A geometry optimization was considered convergent when the force change was smaller than 0.03 eV/Å. Grimme’s DFT-D3 methodology was used to describe the dispersion interactions.^[6]^ The vacuum spacing perpendicular to the plane of the structure is 20 Å. The Brillouin zone integral utilized the surfaces structures of 1×1×1 monkhorst pack K-point sampling. Finally, the adsorption energies (E_ads_) were calculated as E_ads_= E_ad/sub_-E_ad_-E_sub_, where E_ad/sub_, E_ad_, and E_sub_ are the total energies of the optimized adsorbate/substrate system, the adsorbate in the structure, and the clean substrate, respectively. The free energy was calculated using the equation:

G=E_ads_+ZPE-TS

where G, E_ads_, ZPE and TS are the free energy, total energy from DFT calculations, zero point energy and entropic contributions, respectively.


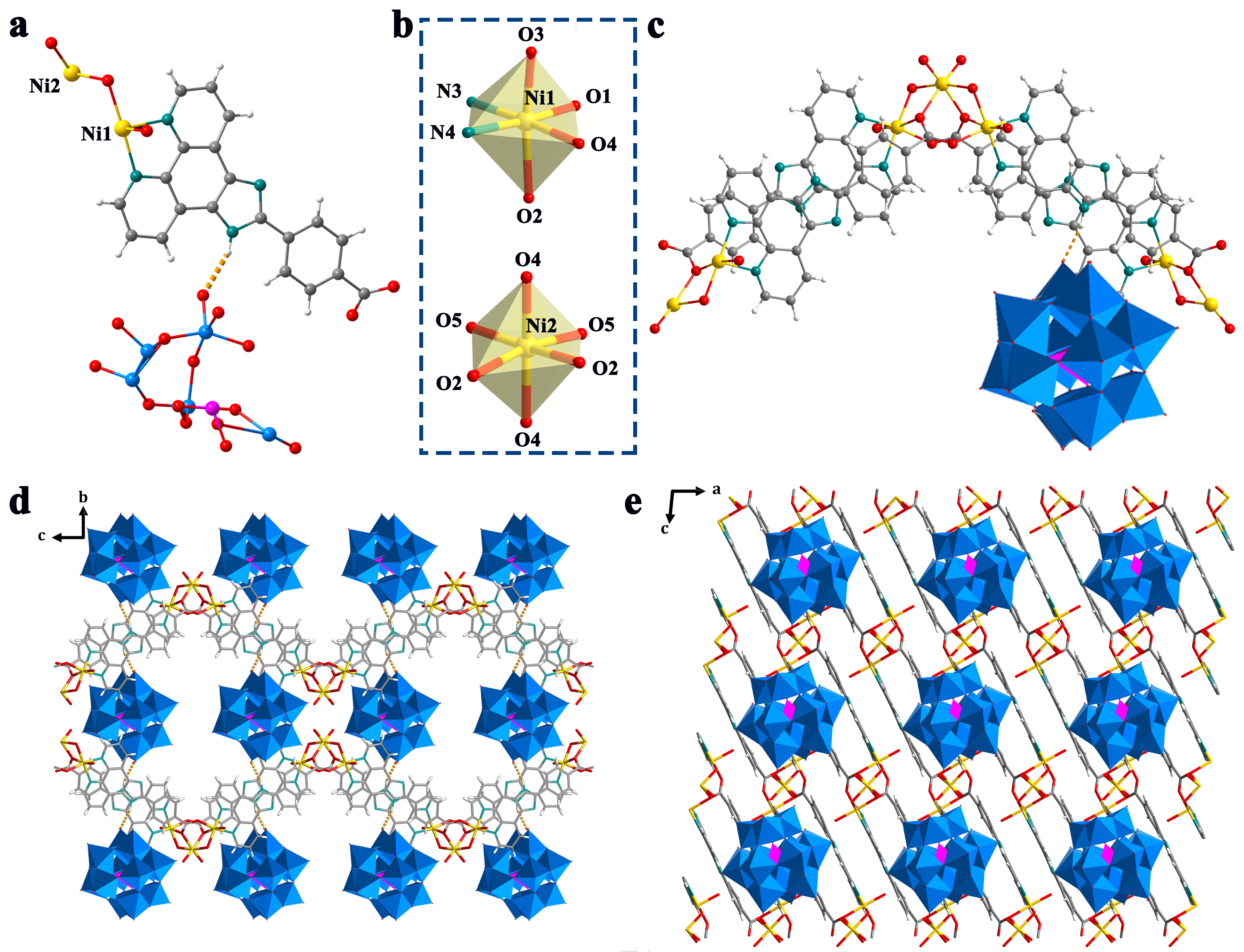


**Figure S1.** a) The asymmetric unit of Ni-PW_12_-1D. b) The coordination mode of Ni ion. c) The basic crystallographic unit in Ni-PW_12_-1D. The 3D framework of Ni-PW_12_-1D along the d) a axis and e) b axis.


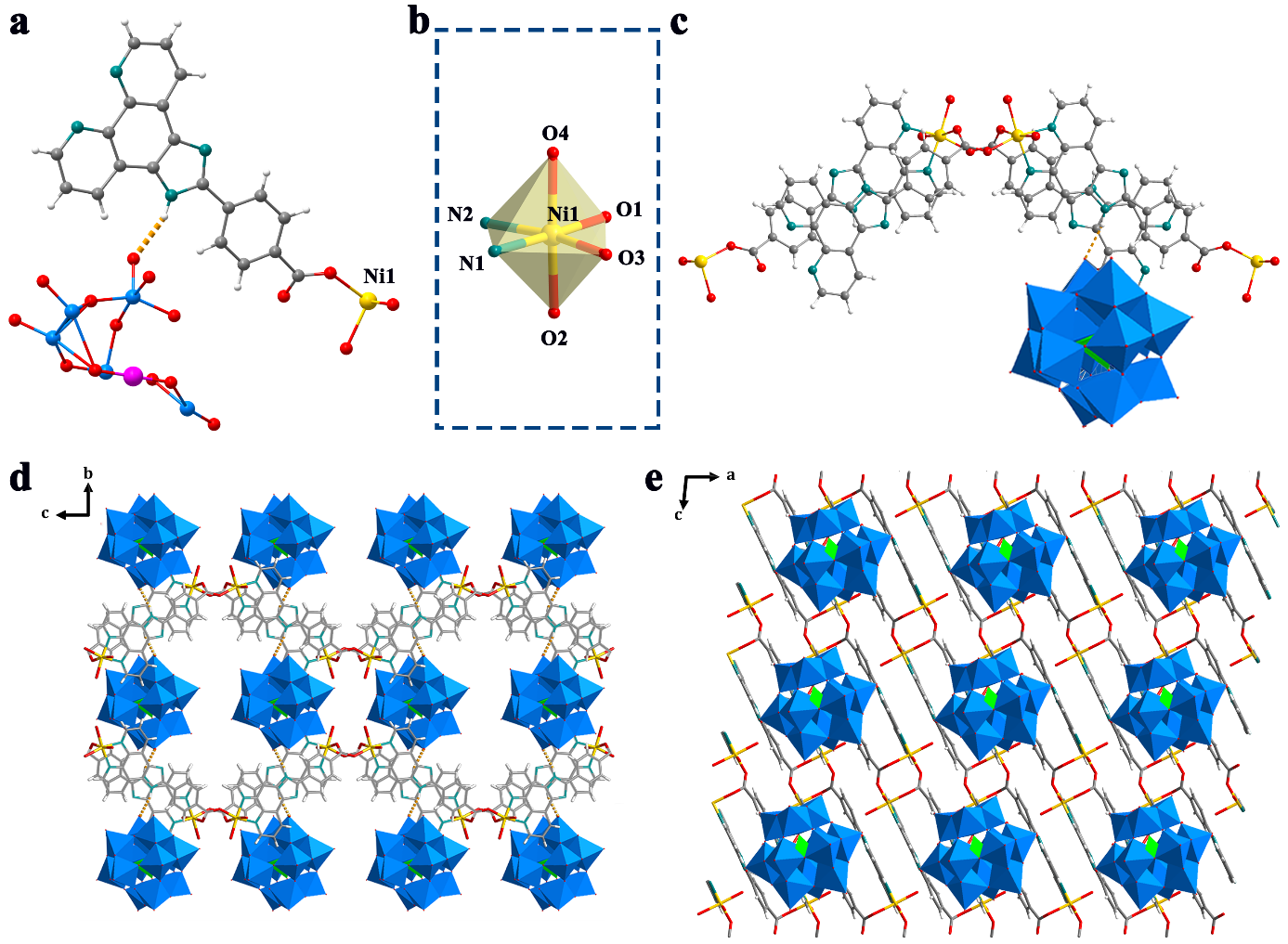


**Figure S2.** a) The asymmetric unit of Ni-SiW_12_-1D. b) The coordination mode of Ni ion. c) The basic crystallographic unit in Ni-SiW_12_-1D. The 3D framework of Ni-SiW_12_-1D along the d) a axis and e) b axis.


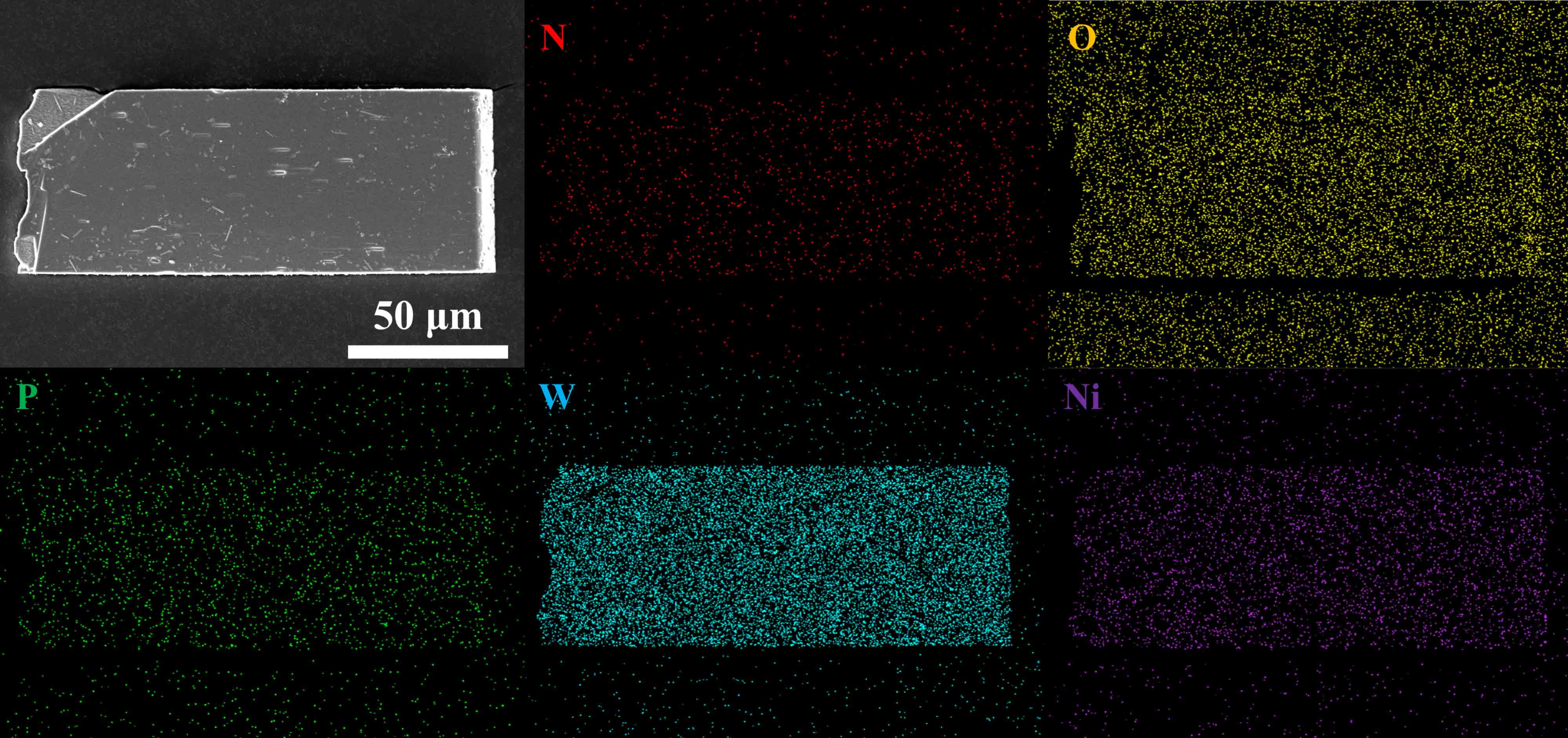


**Figure S3.** SEM and corresponding EDS mapping images of Ni-PW_12_-1D.


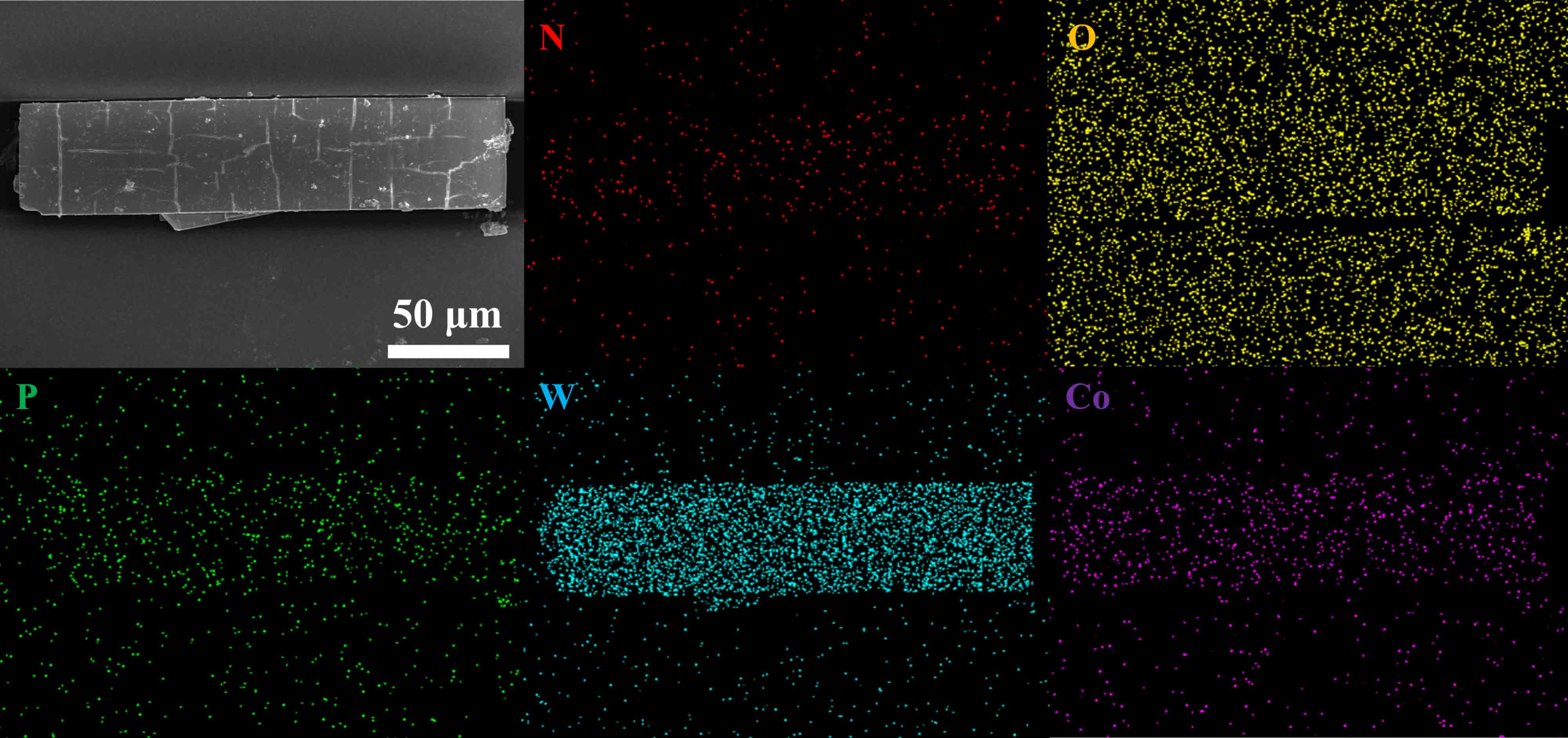


**Figure S4.** SEM and corresponding EDS mapping images of Co-PW_12_-1D.


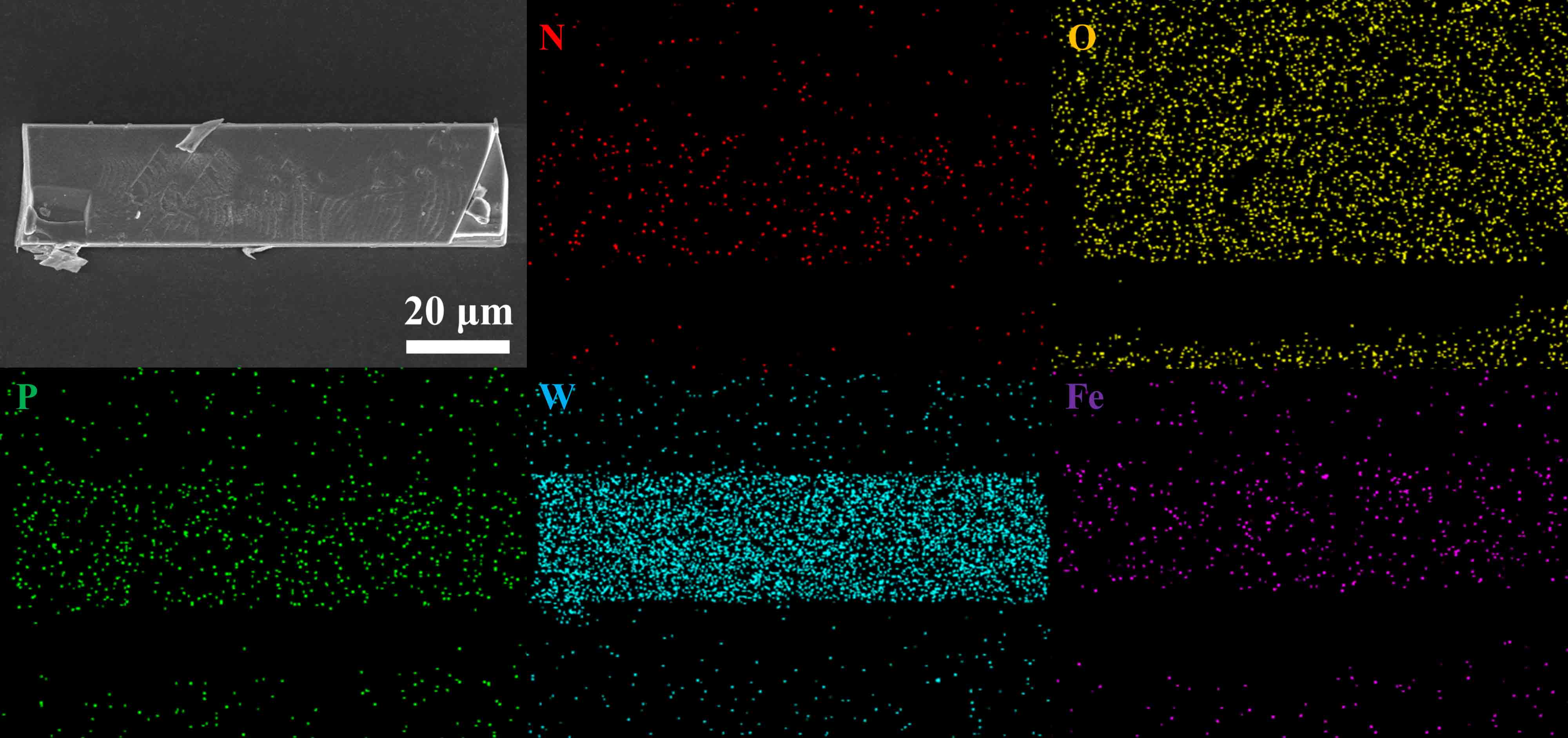


**Figure S5.** SEM and corresponding EDS mapping images of Fe-PW_12_-1D.


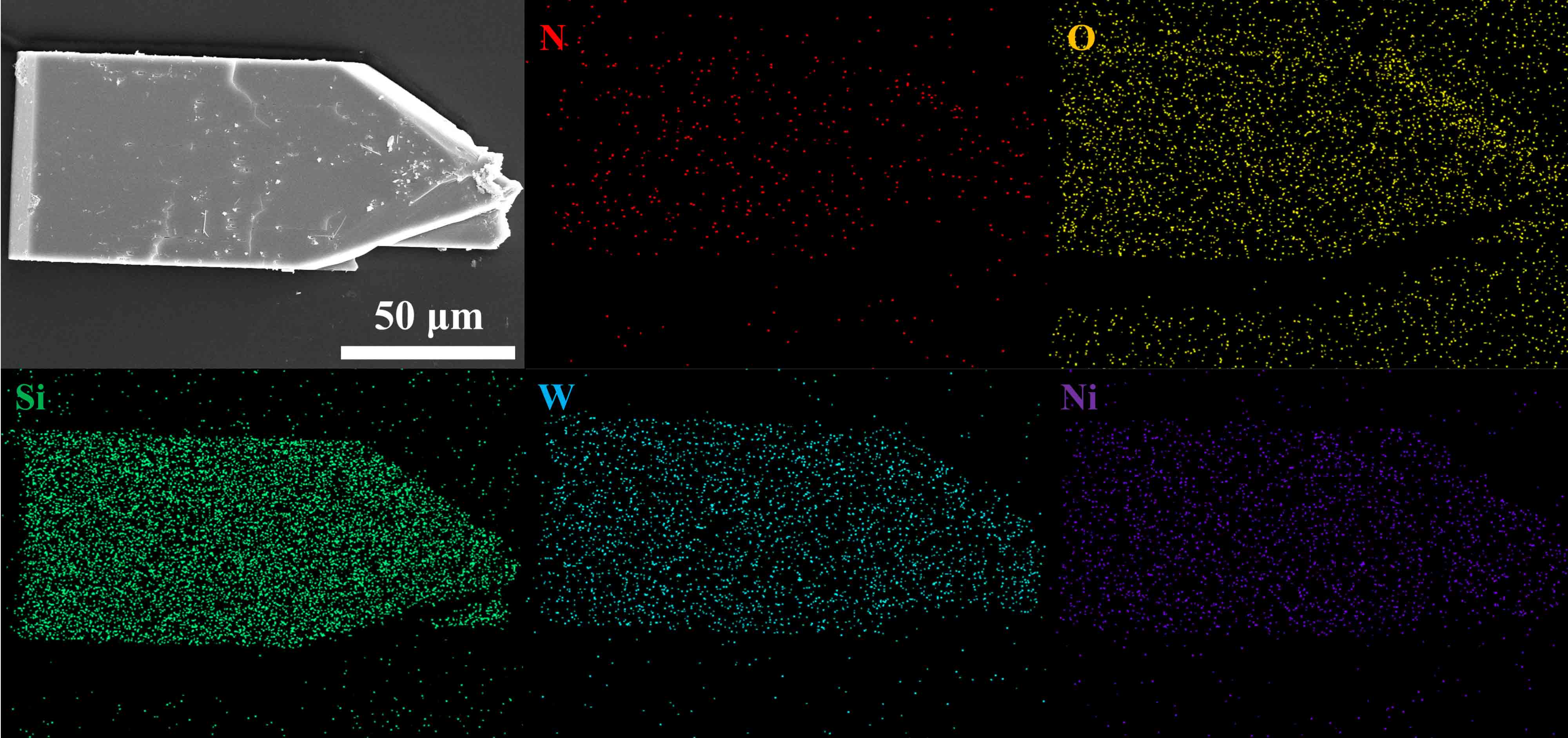


**Figure S6.** SEM and corresponding EDS mapping images of Ni-SiW_12_-1D.


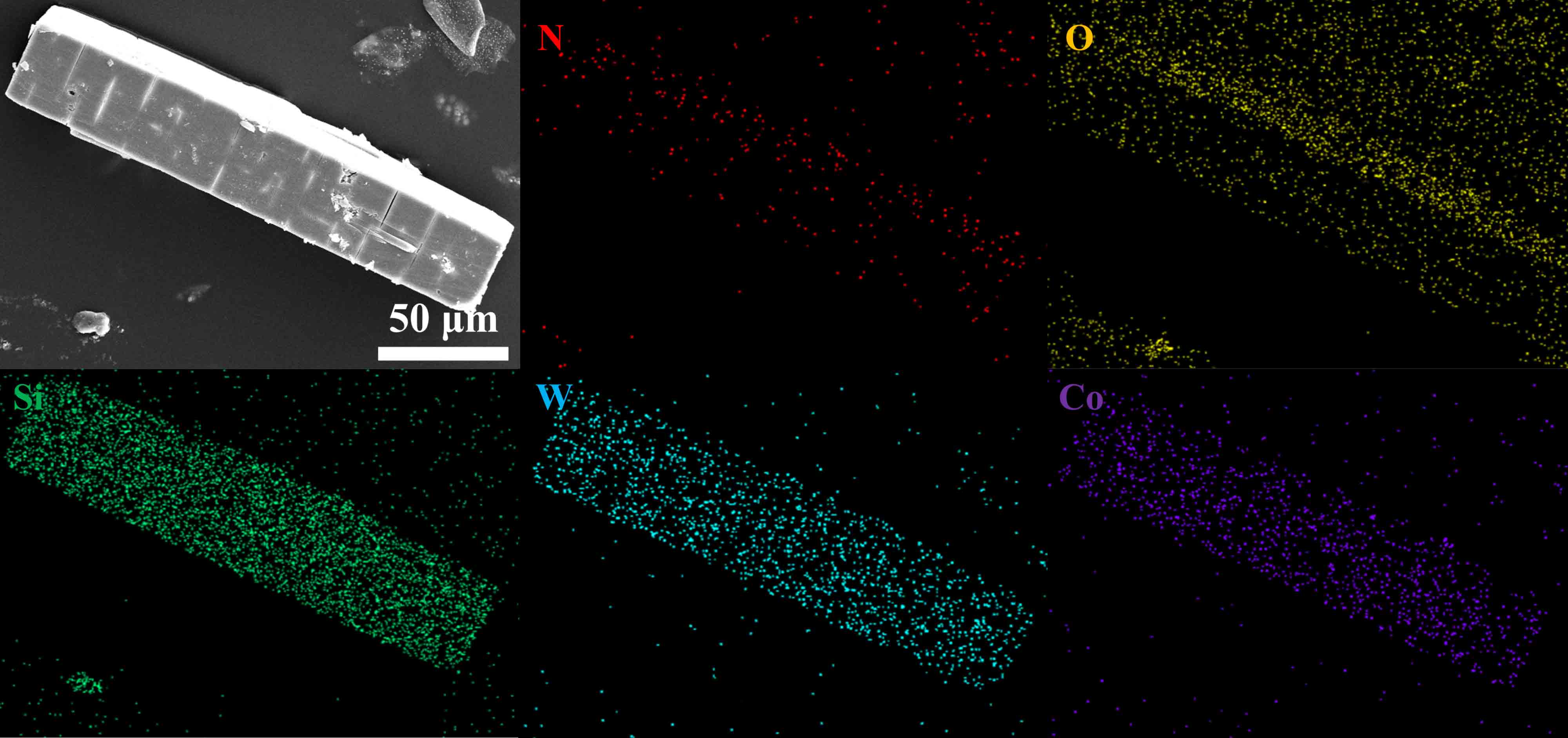


**Figure S7.** SEM and corresponding EDS mapping images of Co-SiW_12_-1D.


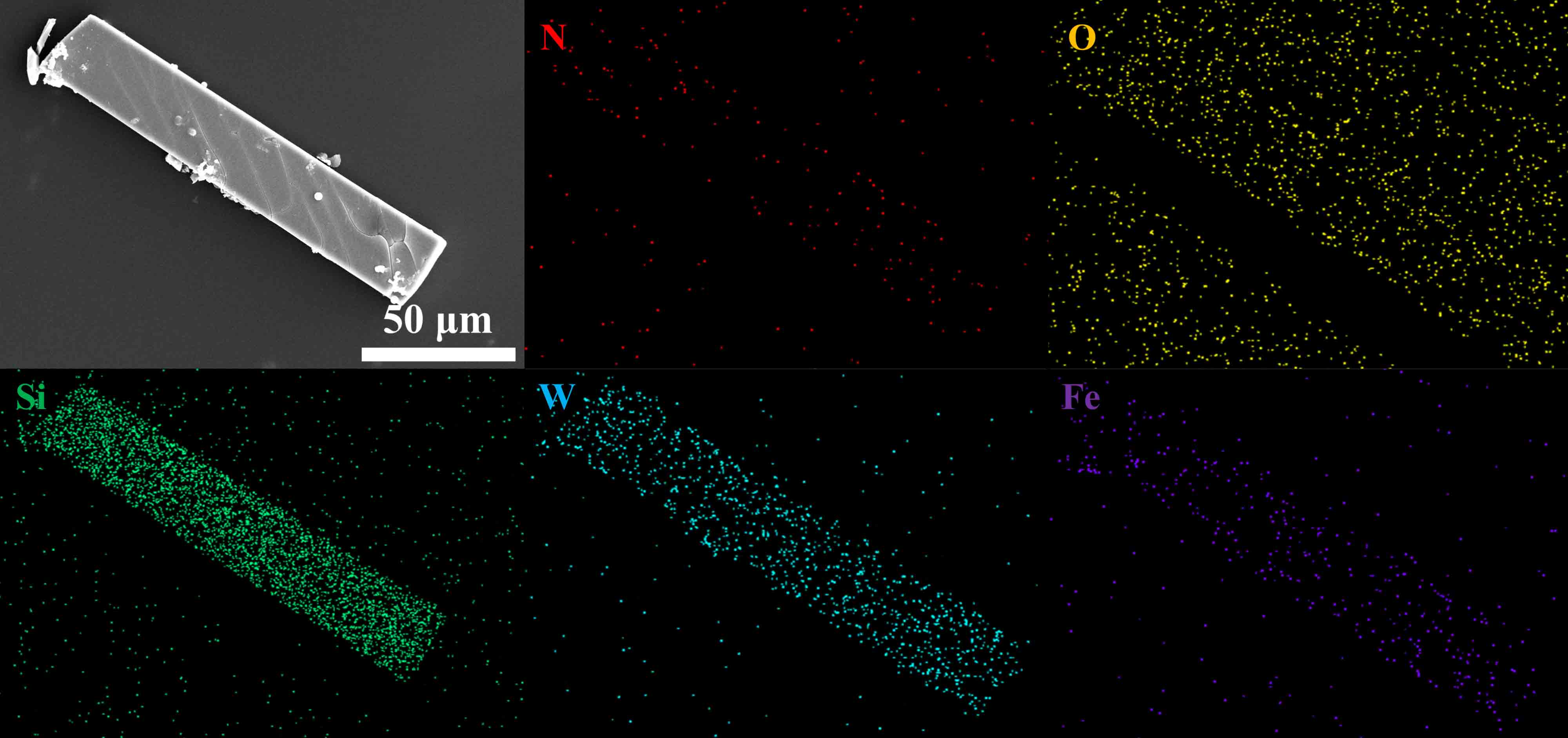


**Figure S8.** SEM and corresponding EDS mapping images of Fe-SiW_12_-1D.


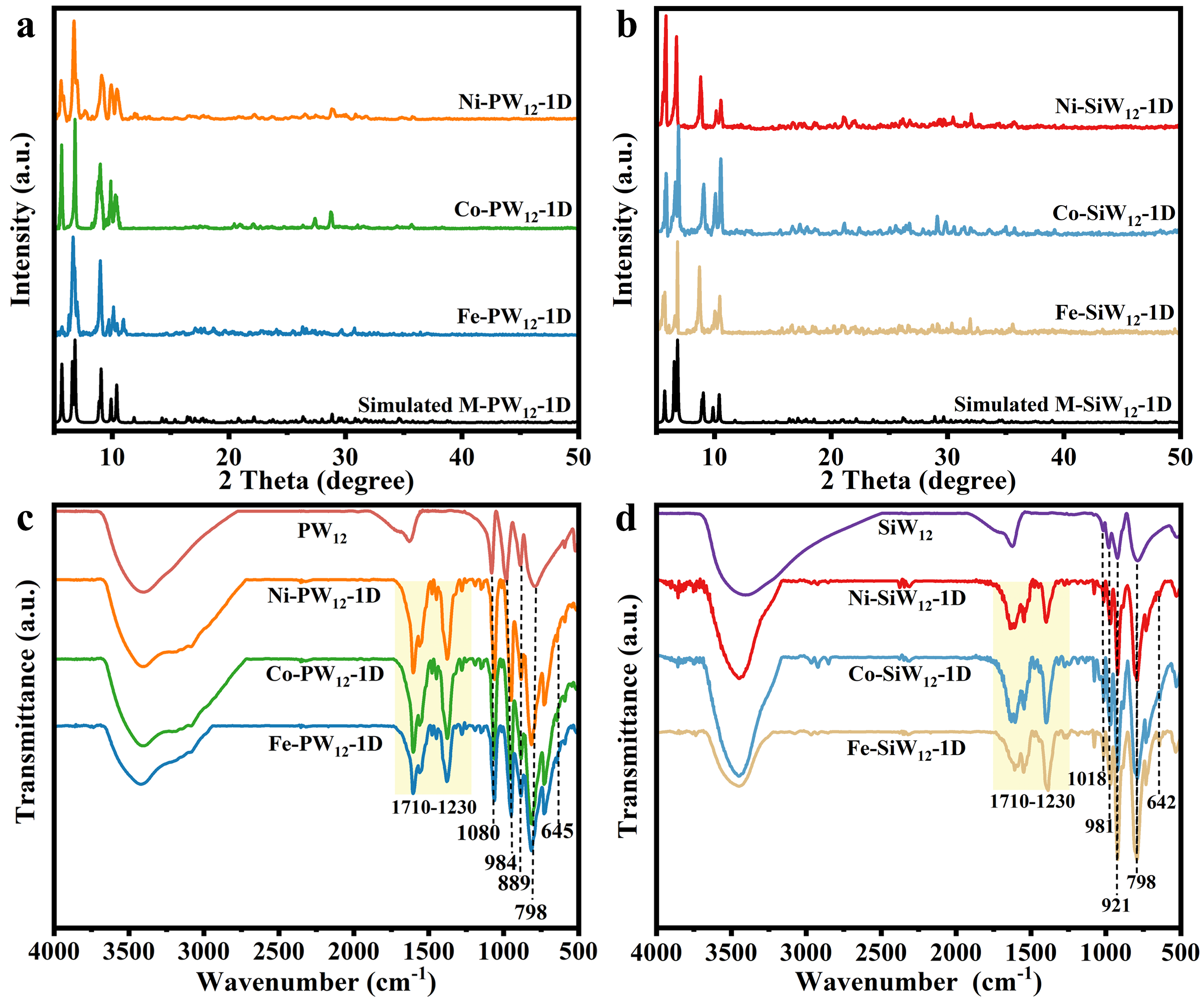


**Figure S9.** PXRD patterns of a) PW_12_ and M-PW_12_-1D; b) SiW_12_ and M-SiW_12_-1D.


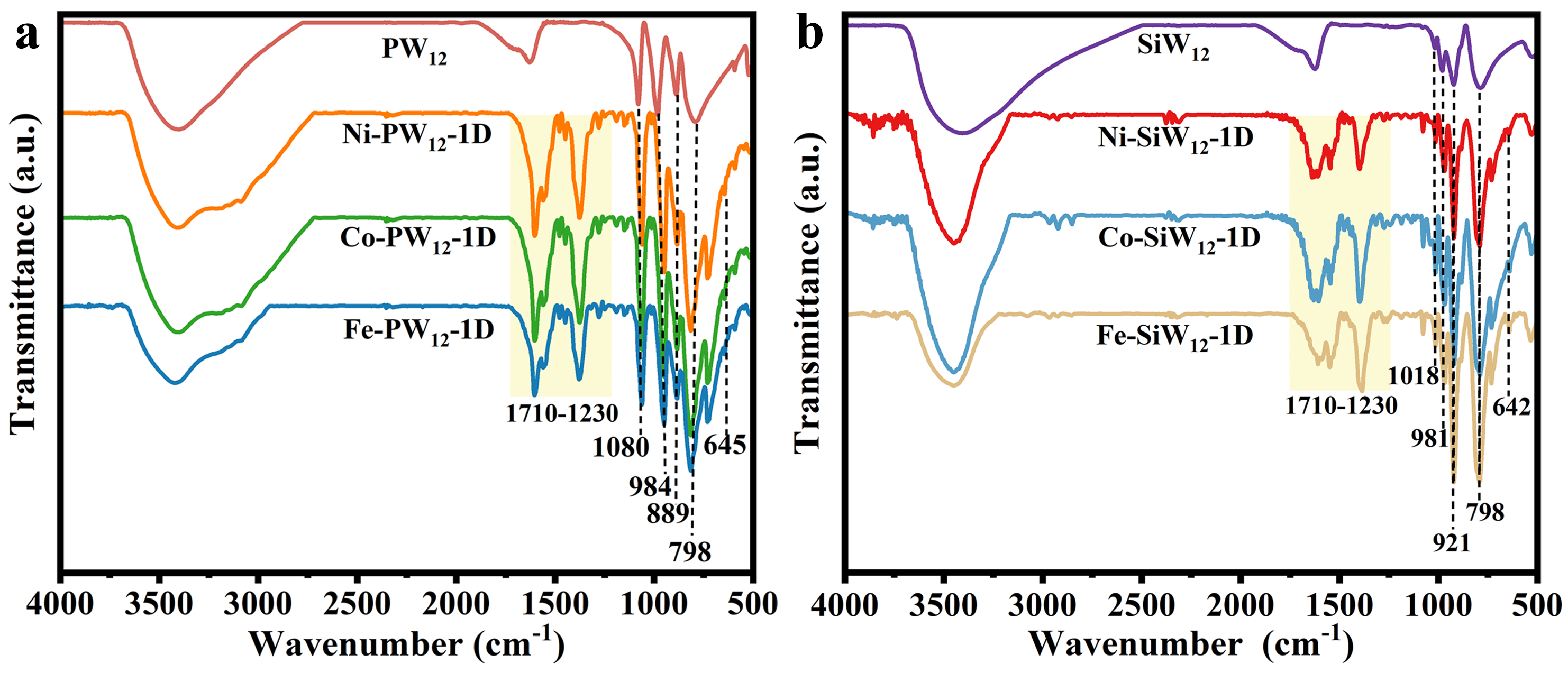


**Figure S10.** FTIR spectra of a) PW_12_ and M-PW_12_-1D; b) SiW_12_ and M-SiW_12_-1D.


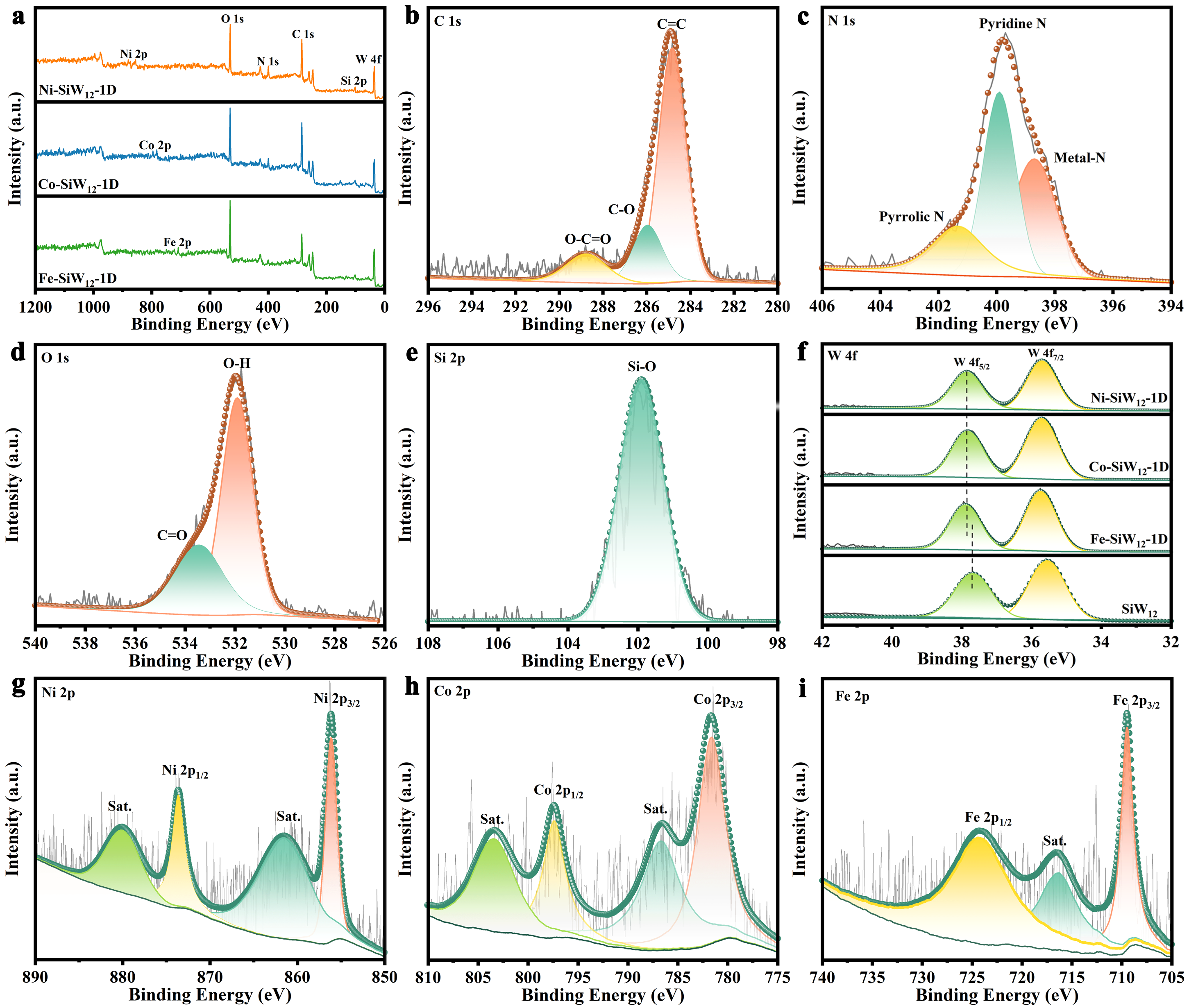


**Figure S11.** XPS spectra of Ni-SiW_12_-1D: a) survey, b) C 1s, c) N 1s, d) O 1s and e) Si 2p. f) W 4f, g) Ni 2p, h) Co 2p and i) Fe 2p spectra of SiW_12_ and M-SiW_12_-1D.


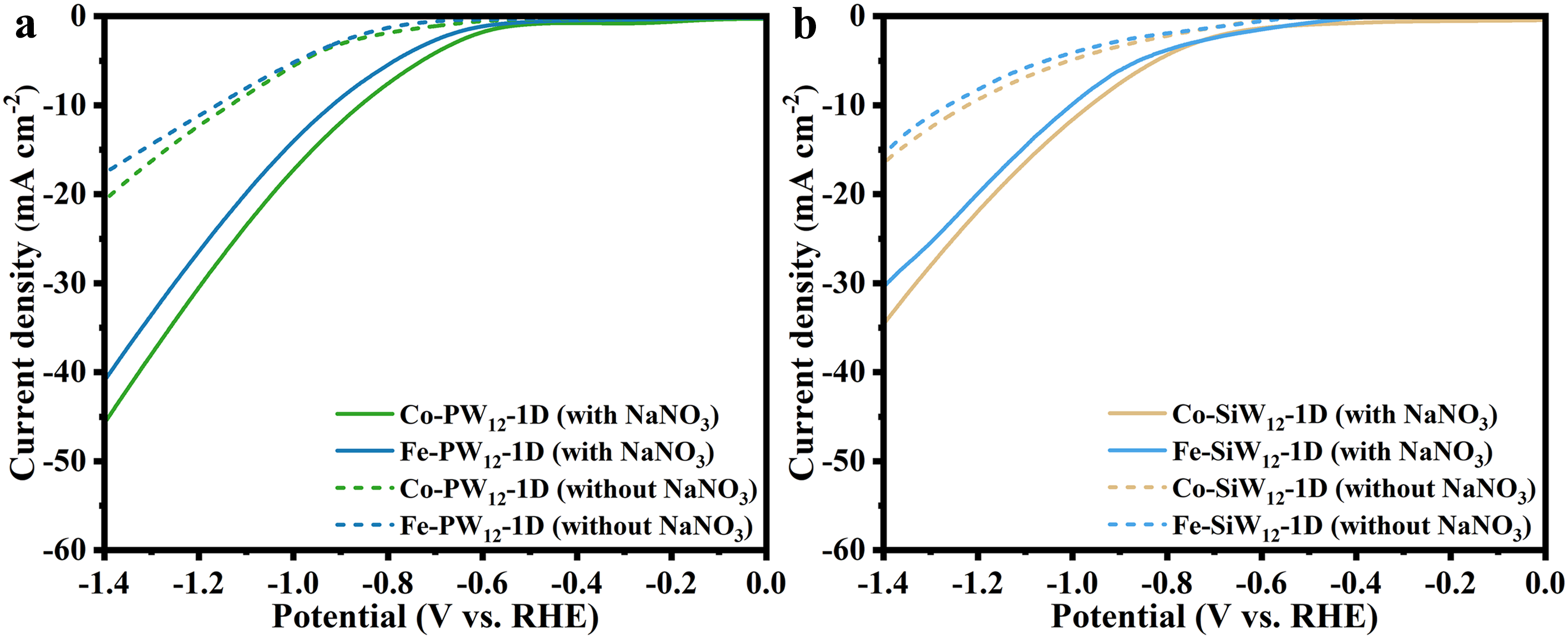


**Figure** **S12.** LSV plots of a) Co-PW_12_-1D and Fe-PW_12_-1D; b) Co-SiW_12_-1D and Fe-SiW_12_-1D, both in the presence and absence of nitrate.


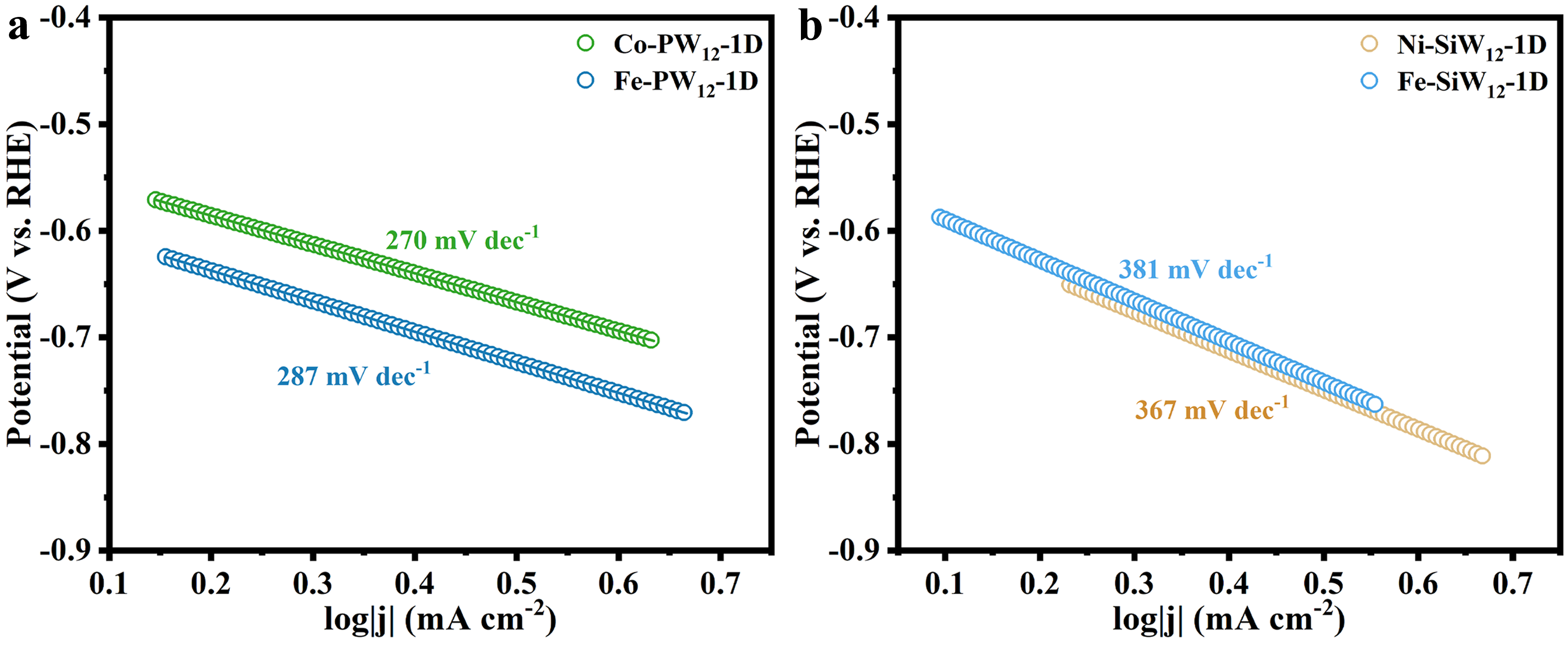


**Figure S13.** Tafel plots of a) Co-PW_12_-1D and Fe-PW_12_-1D; b) Co-SiW_12_-1D and Fe-SiW_12_-1D.


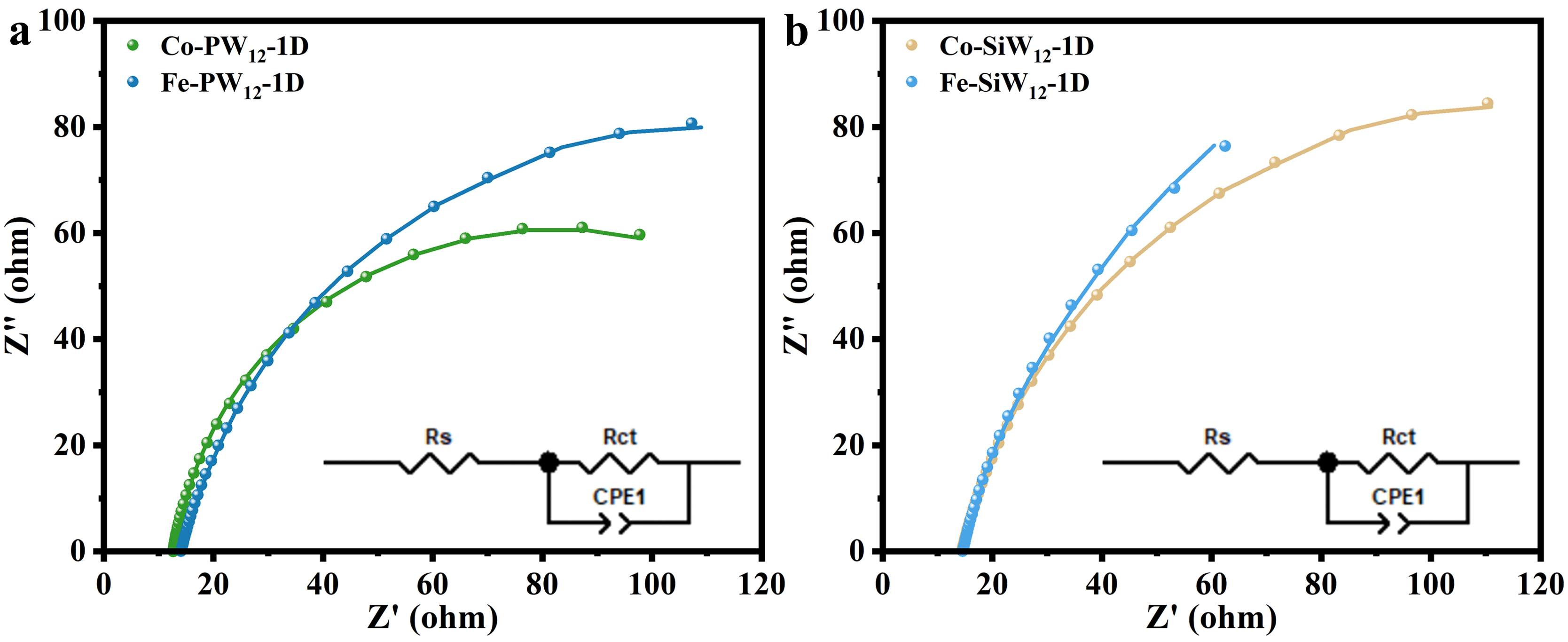


**Figure S14.** EIS spectra in 0.1 M NaNO_3_/0.1 M Na_2_SO_4_: a) Co-PW_12_-1D and Fe-PW_12_-1D; b) Co-SiW_12_-1D and Fe-SiW_12_-1D.


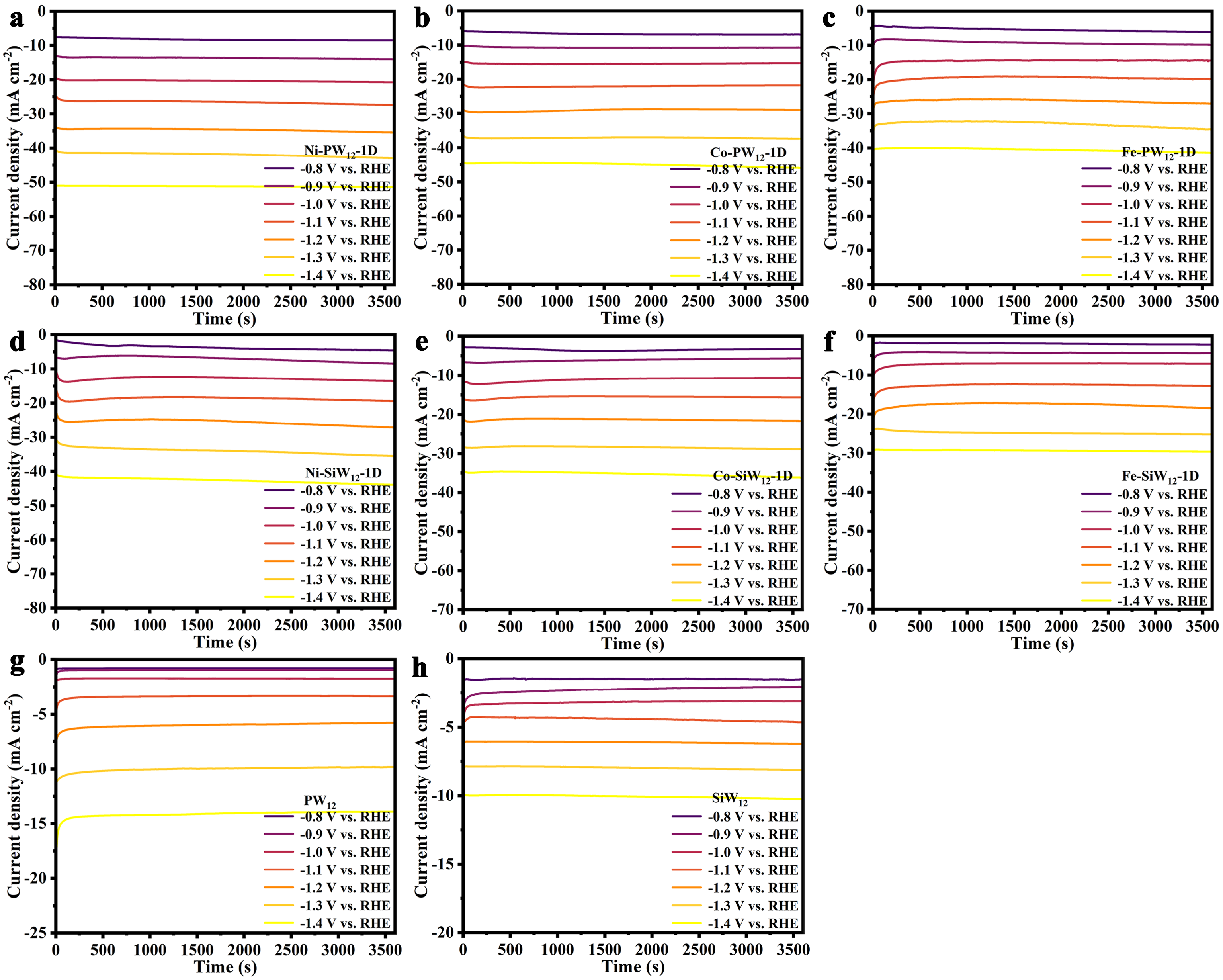


**Figure S15.** The chronoamperometry curves at various potentials of a) Ni-PW_12_-1D_,_ b) Co-PW_12_-1D, c) Fe-PW_12_-1D, d) Ni-SiW_12_-1D_,_ e) Co-SiW_12_-1D, f) Fe-SiW_12_-1D_,_ g) PW_12_ and h) SiW_12_.


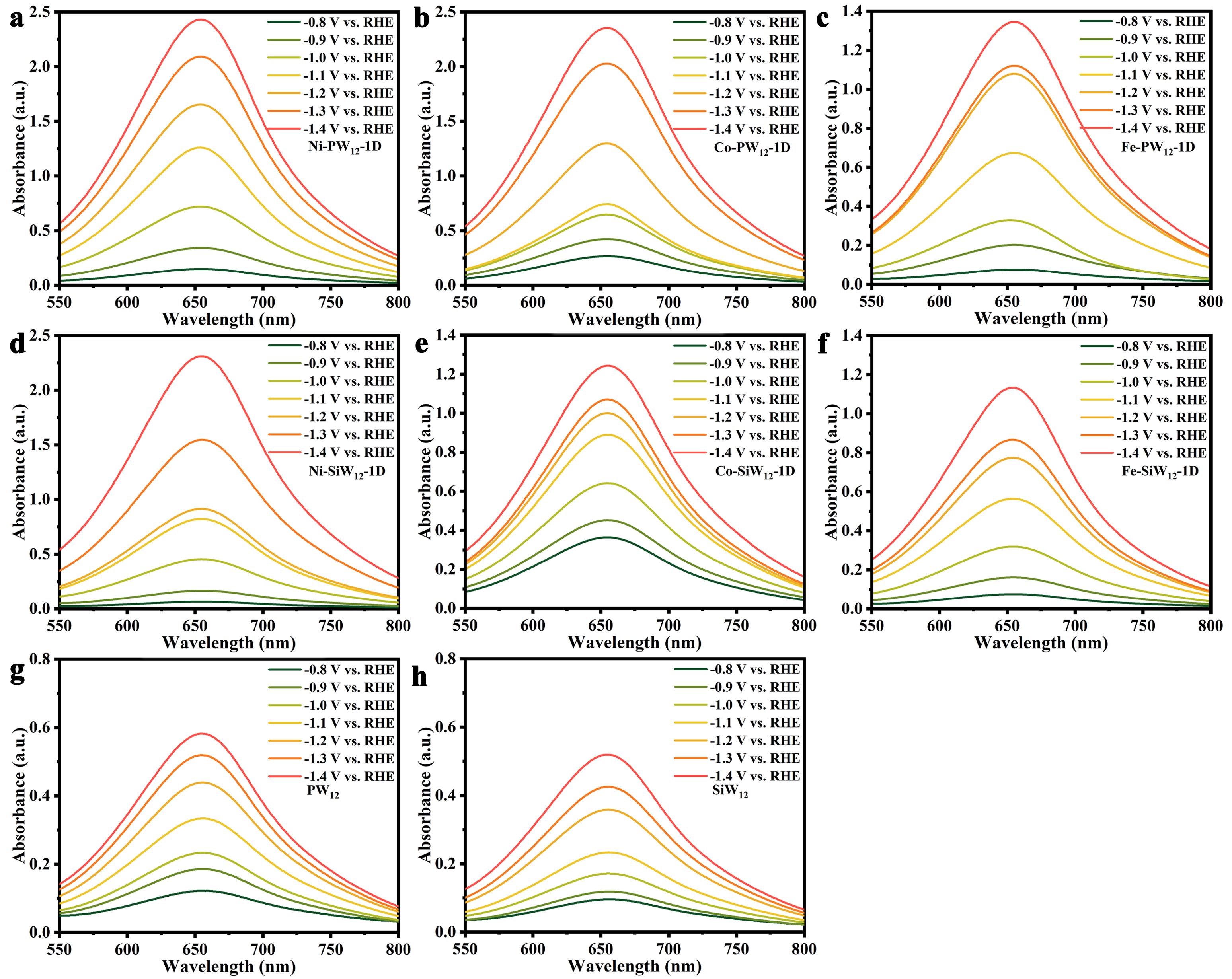


**Figure S16.** The UV-Vis spectra of NH_3_ Generated by a) Ni-PW_12_-1D_,_ b) Co-PW_12_-1D, c) Fe-PW_12_-1D, d) Ni-SiW_12_-1D_,_ e) Co-SiW_12_-1D, f) Fe-SiW_12_-1D_,_ g) PW_12_ and h) SiW_12_ at different potentials in 0.1 M Na_2_SO_4_ with 0.1 M NaNO_3_.


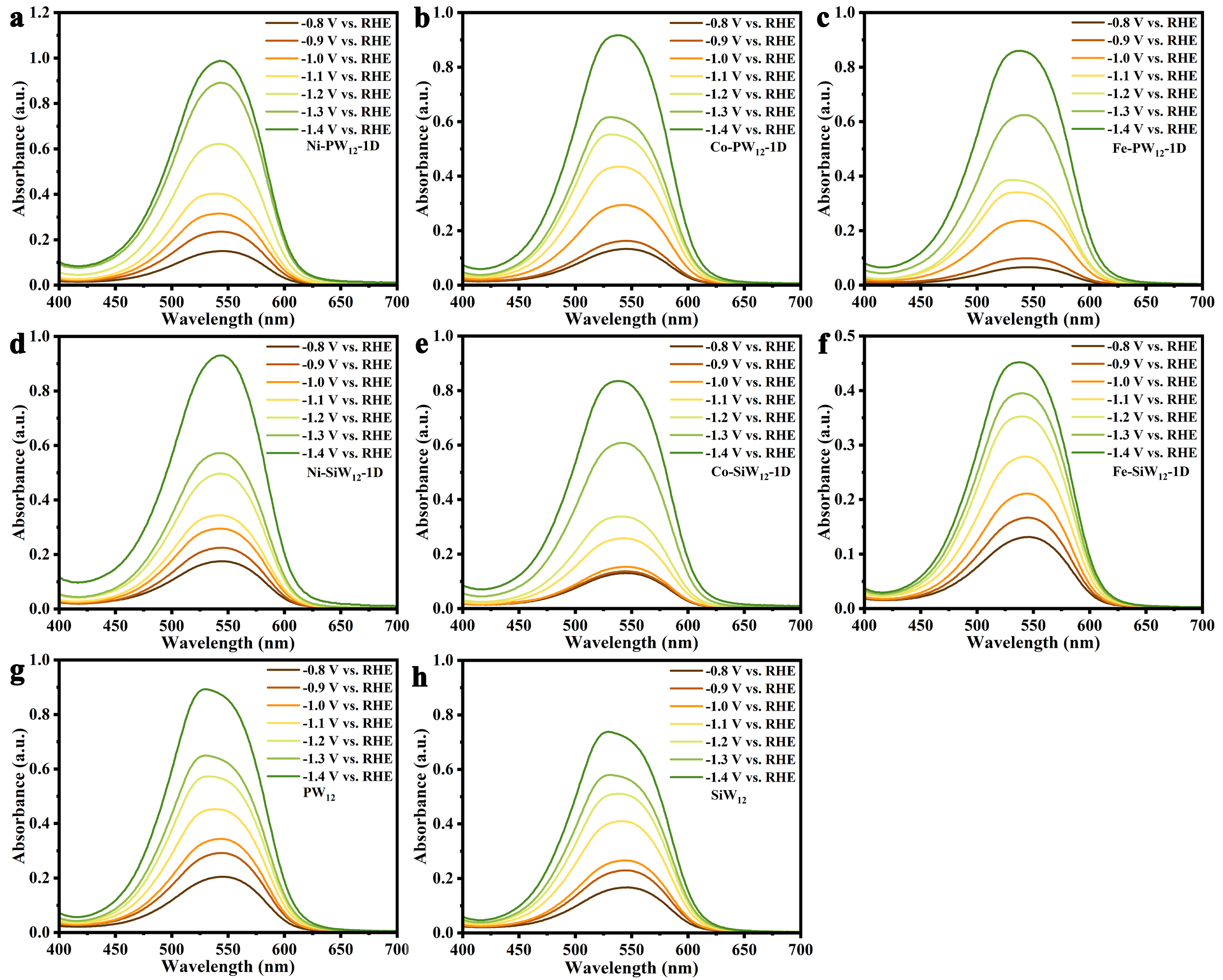


**Figure S17.** The UV-Vis spectra of NO_2_^–^ Generated by a) Ni-PW_12_-1D_,_ b) Co-PW_12_-1D, c) Fe-PW_12_-1D, d) Ni-SiW_12_-1D_,_ e) Co-SiW_12_-1D, f) Fe-SiW_12_-1D_,_ g) PW_12_ and h) SiW_12_ at different potentials in 0.1 M Na_2_SO_4_ with 0.1 M NaNO_3_.


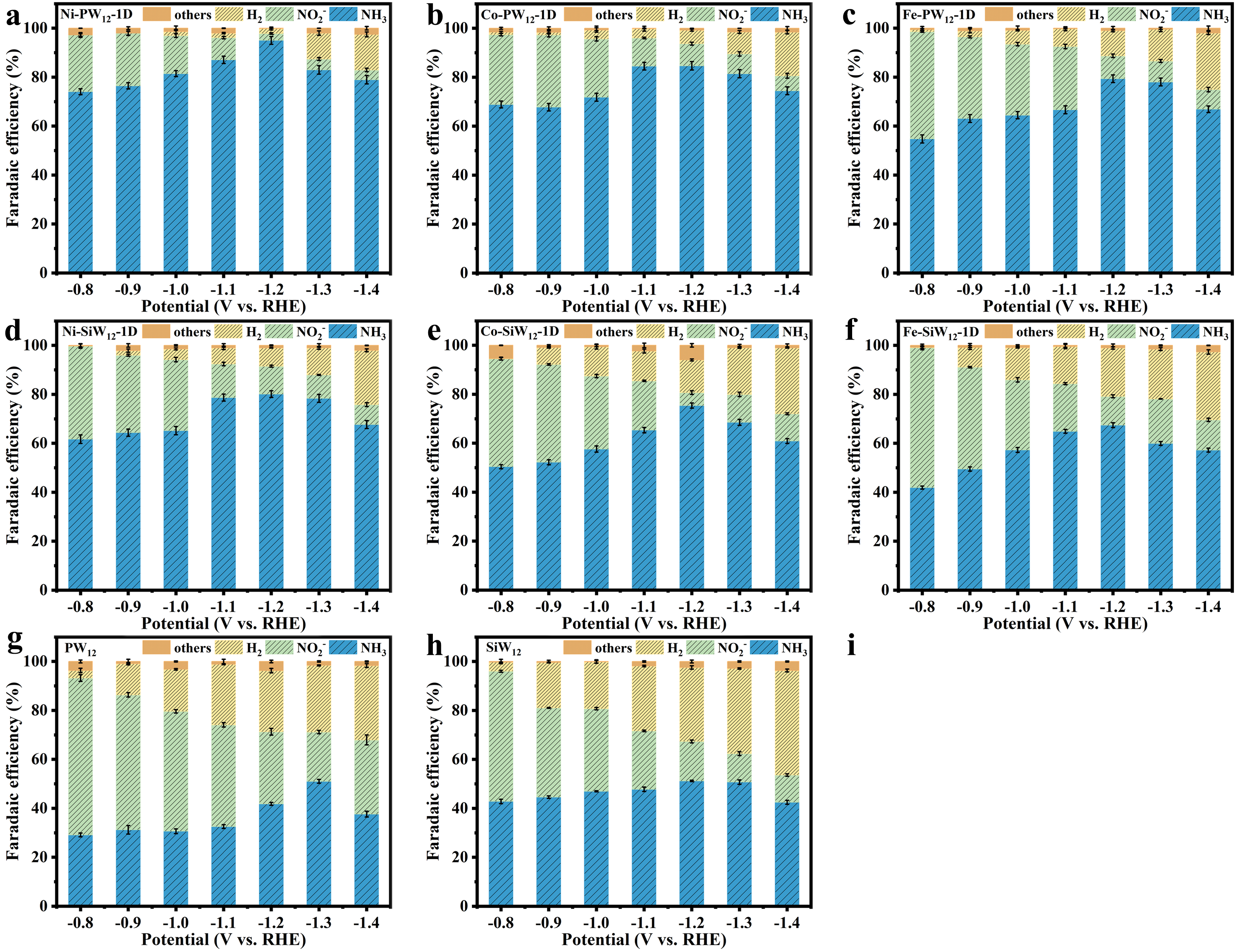


**Figure S18.** FEs of NH_3_, NO_2_^–^, H_2_ and others for a) Ni-PW_12_-1D_,_ b) Co-PW_12_-1D, c) Fe-PW_12_-1D, d) Ni-SiW_12_-1D_,_ e) Co-SiW_12_-1D, f) Fe-SiW_12_-1D_,_ g) PW_12_ and h) SiW_12_ at different potentials in 0.1 M Na_2_SO_4_ with 0.1 M NaNO_3_ (three times error statistics).





**Figure S19.** Maximum FEs of Ni-PW_12_ with concentrations of NO_3_^-^ ranging from 10 to 100 mM (three times error statistics)


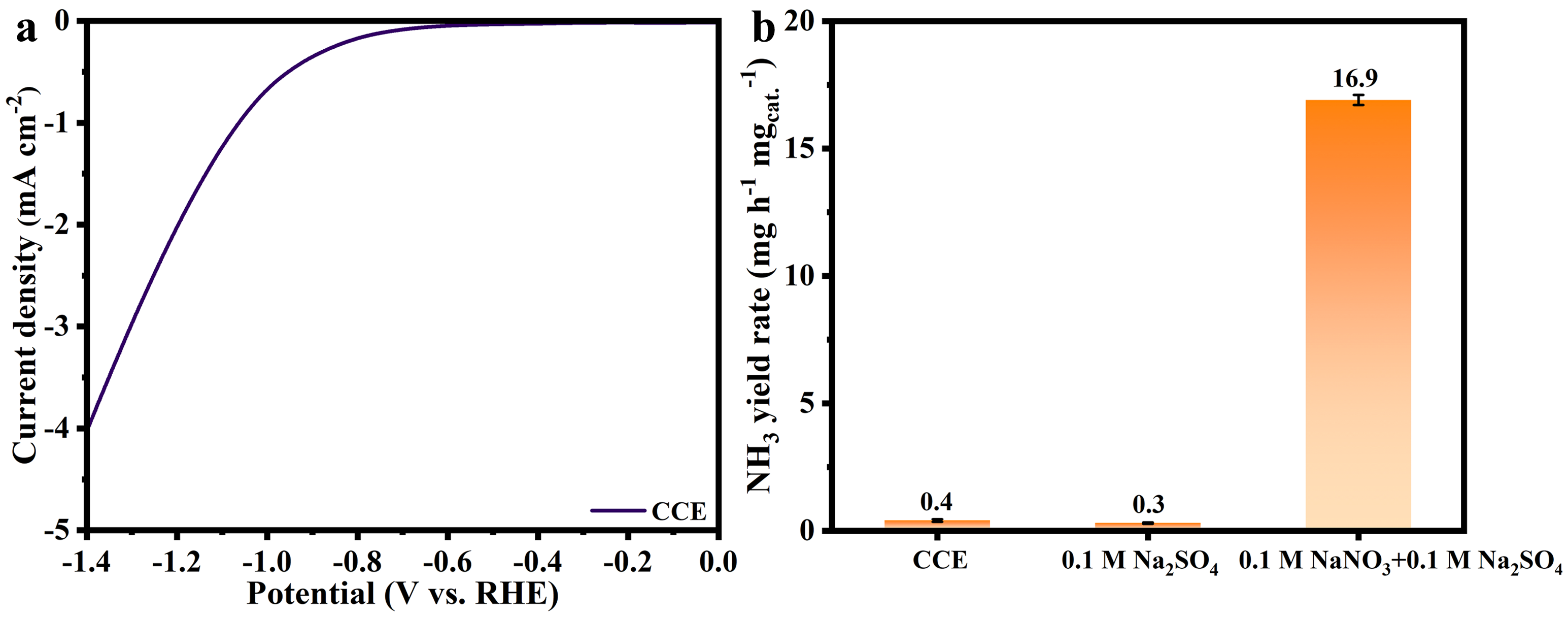


**Figure S20.** a) LSV curve of ENRA catalyzed by CCE as working electrode. b) The NH_3_ yield rates and FEs (at -1.2 V vs. RHE) of ENRA catalyzed by different materials as working electrodes (three times error statistics).


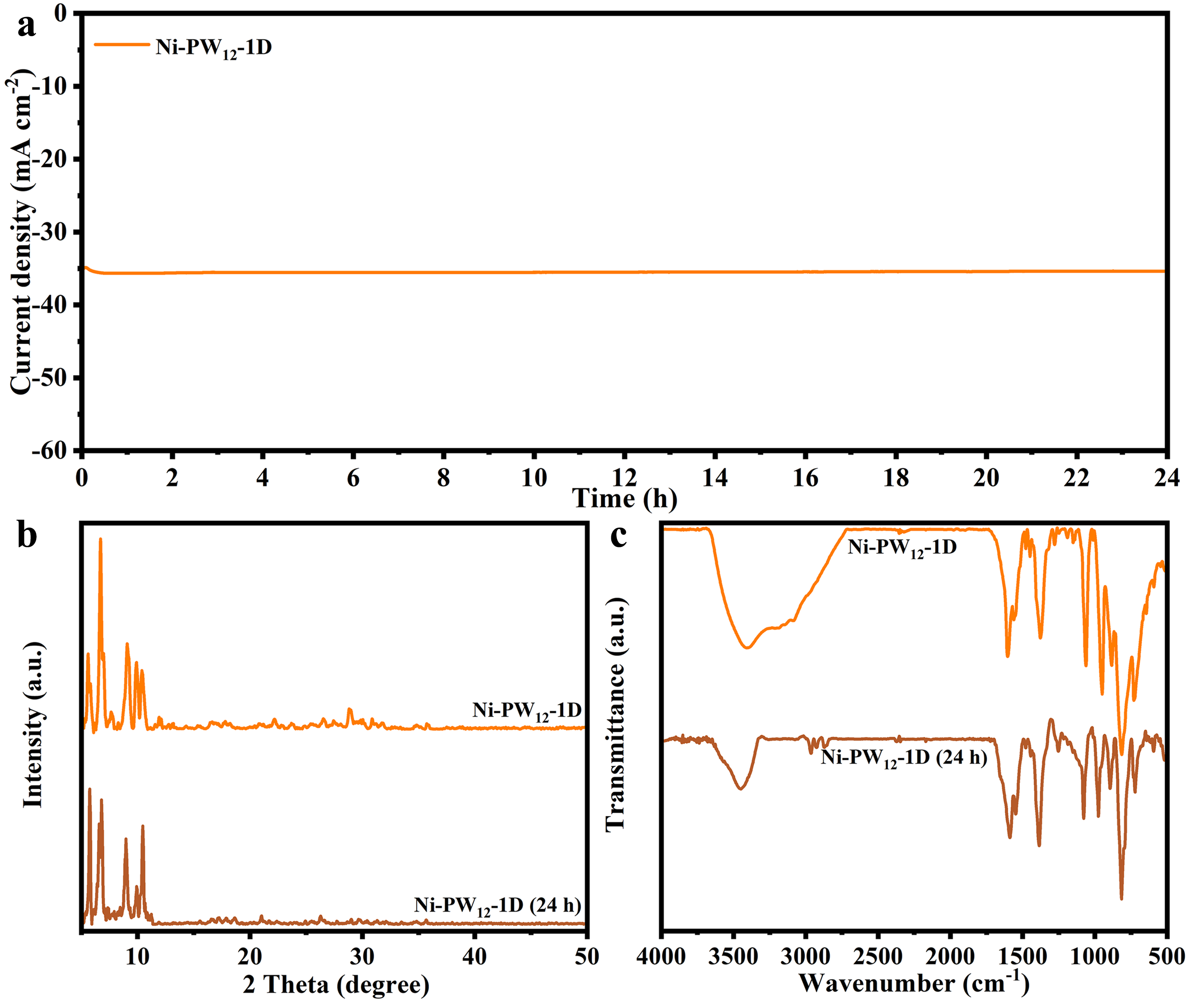


**Figure S21.** a) The i-t curve of Ni-PW_12_-1D at the optimal potential. b) PXRD patterns and c) FTIR spectra of Ni-PW_12_-1D after 24 h.


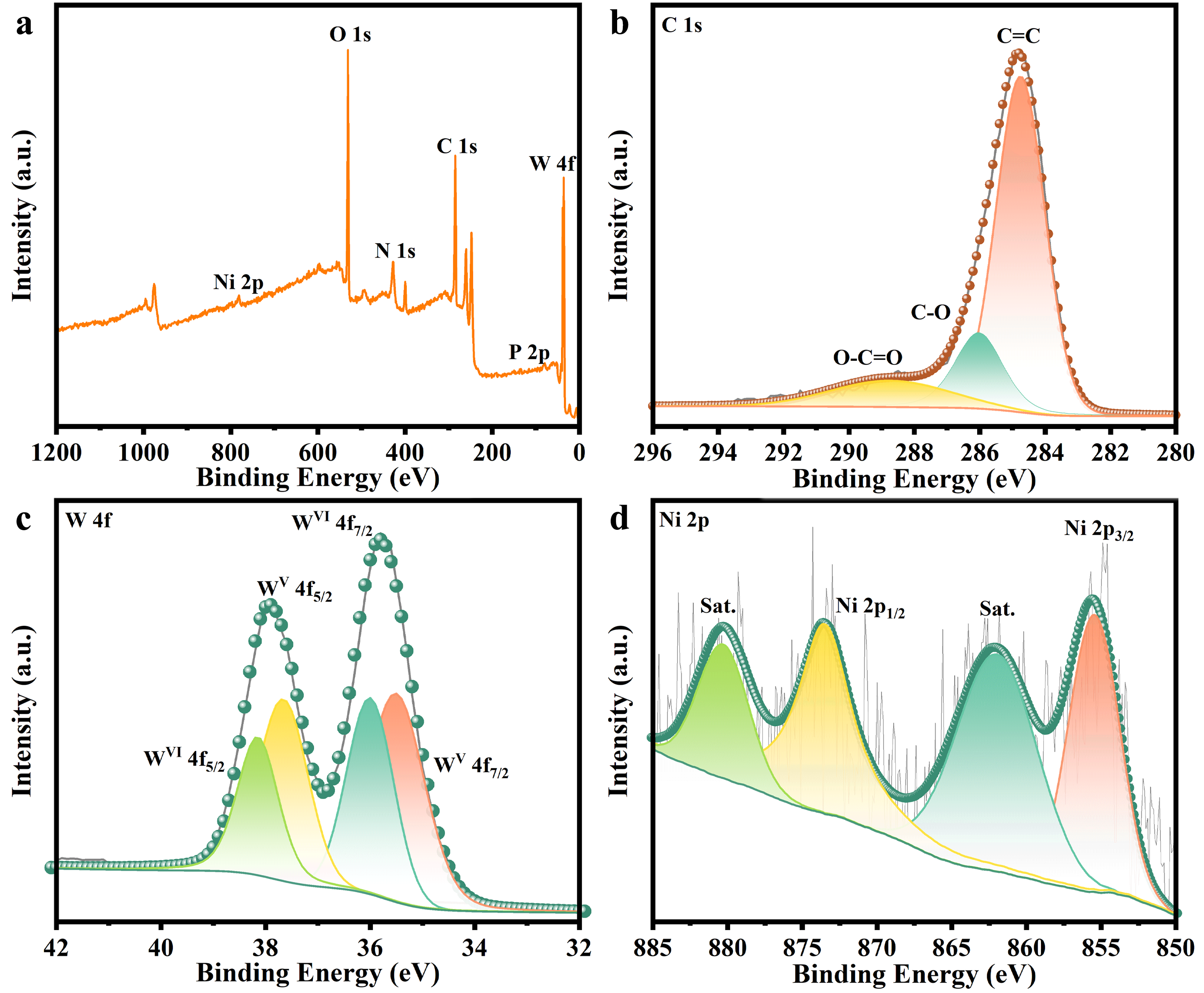


**Figure S22.** a) XPS survey spectra of Ni-PW_12_-1D after 24 h. High-resolution XPS spectra of b) C 1s, c) W 4f and d) Ni 2p spectra for Ni-PW_12_-1D after 24 h.


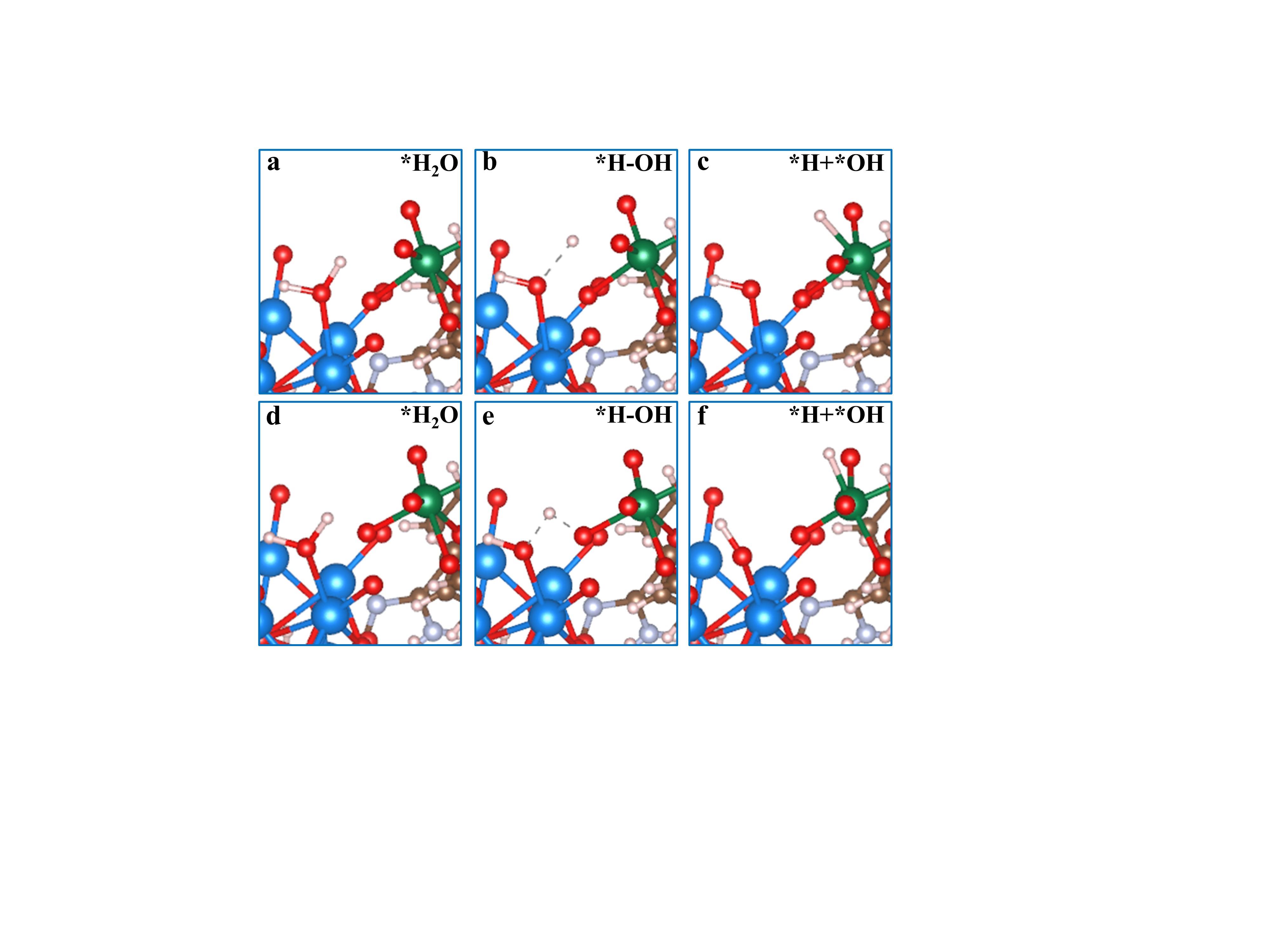


**Figure S23.** The side view structures of intermediates adsorption in water splitting process on the surface of a-c) Ni-PW_12_-1D, d-f) Ni-SiW_12_-1D, respectively.


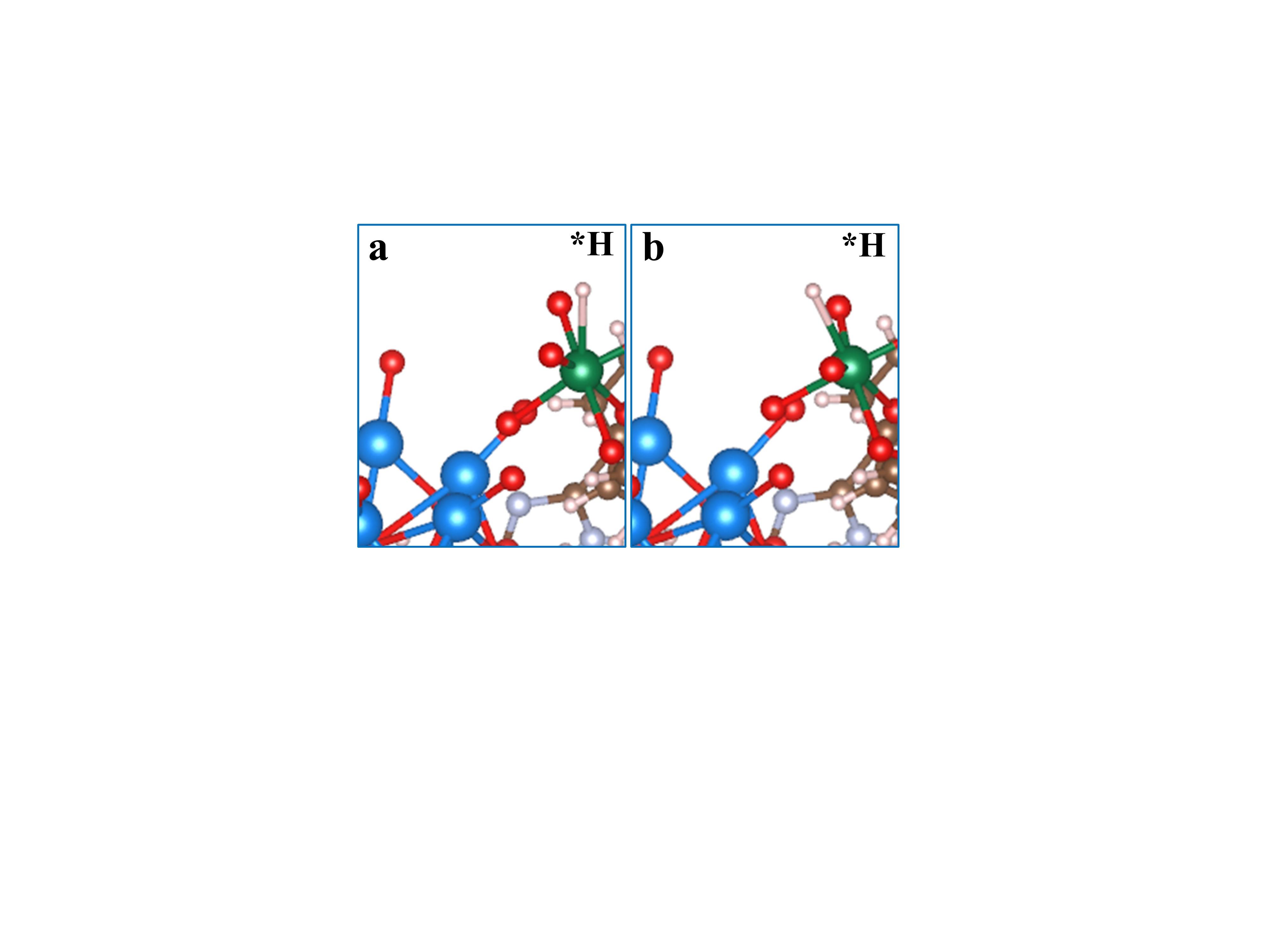


**Figure S24.** The side view structures of *H adsorption in HER process on the surface of a) Ni-PW_12_-1D and b) Ni-SiW_12_-1D.


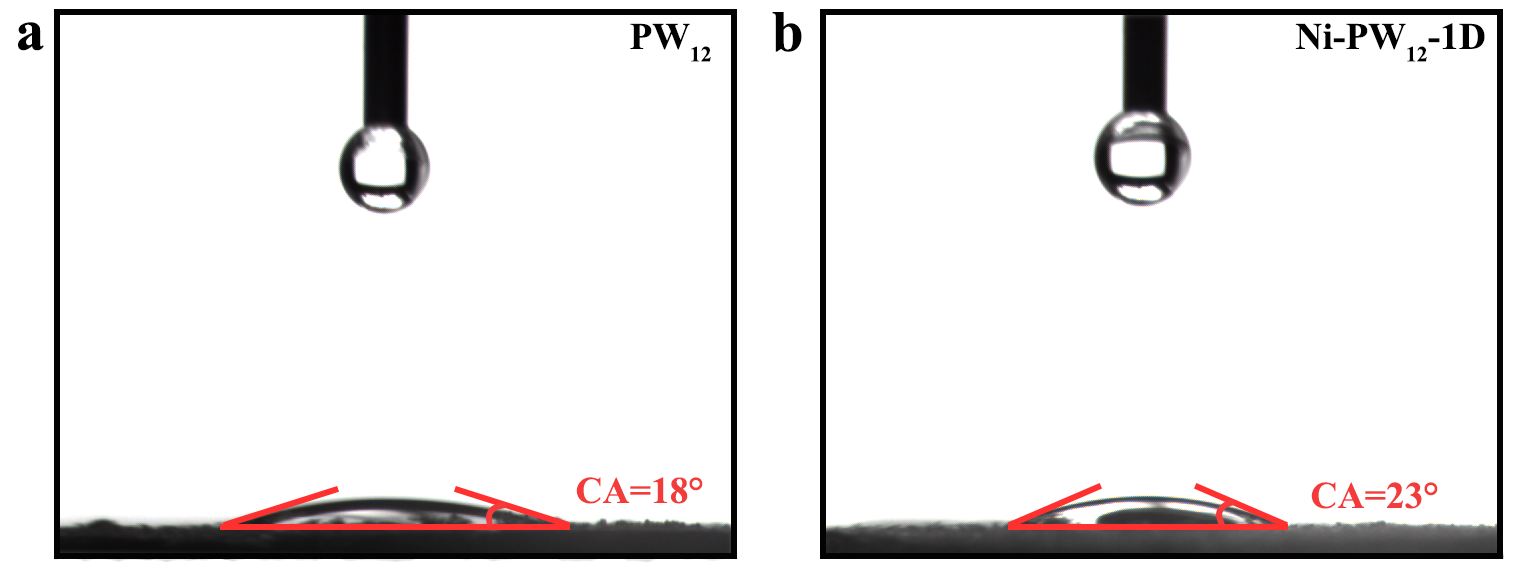


**Figure S25.** The contact angle test results of a) PW_12_ and b) Ni-PW_12_-1D.


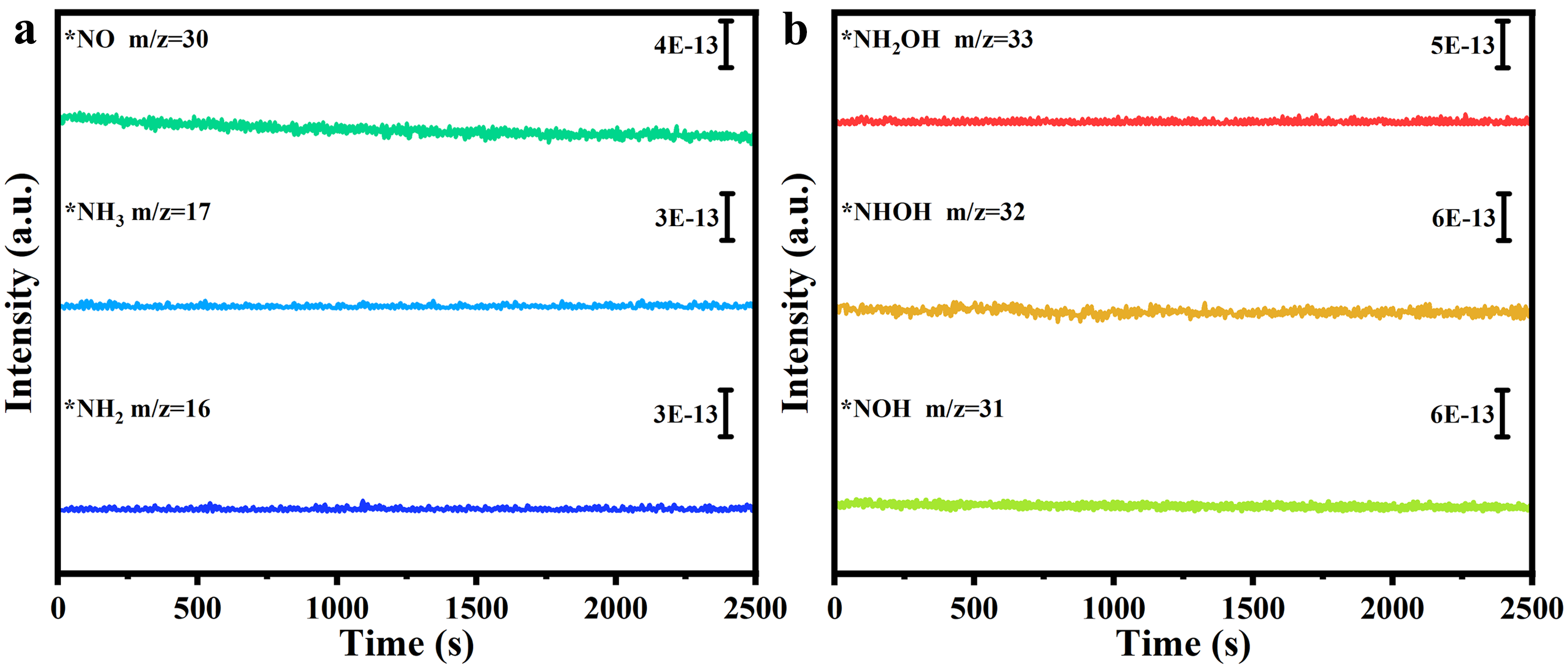


**Figure S26.** a, b) Electrochemical online DEMS results for Ni-PW_12_-1D in the absence of NaNO_3_.


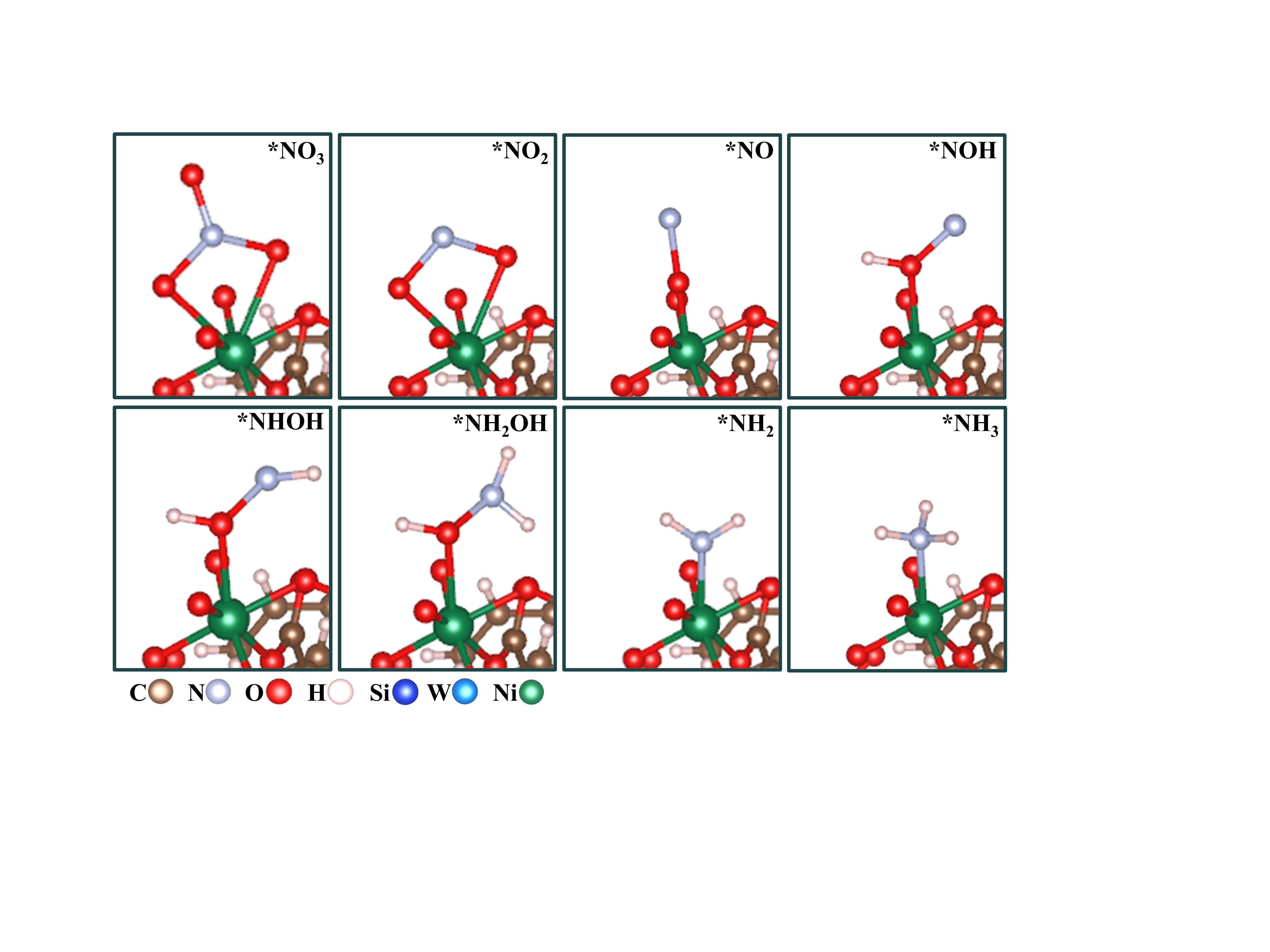


**Figure S27.** DFT results. The adsorption configurations of intermediates in Ni-SiW_12_-1D.


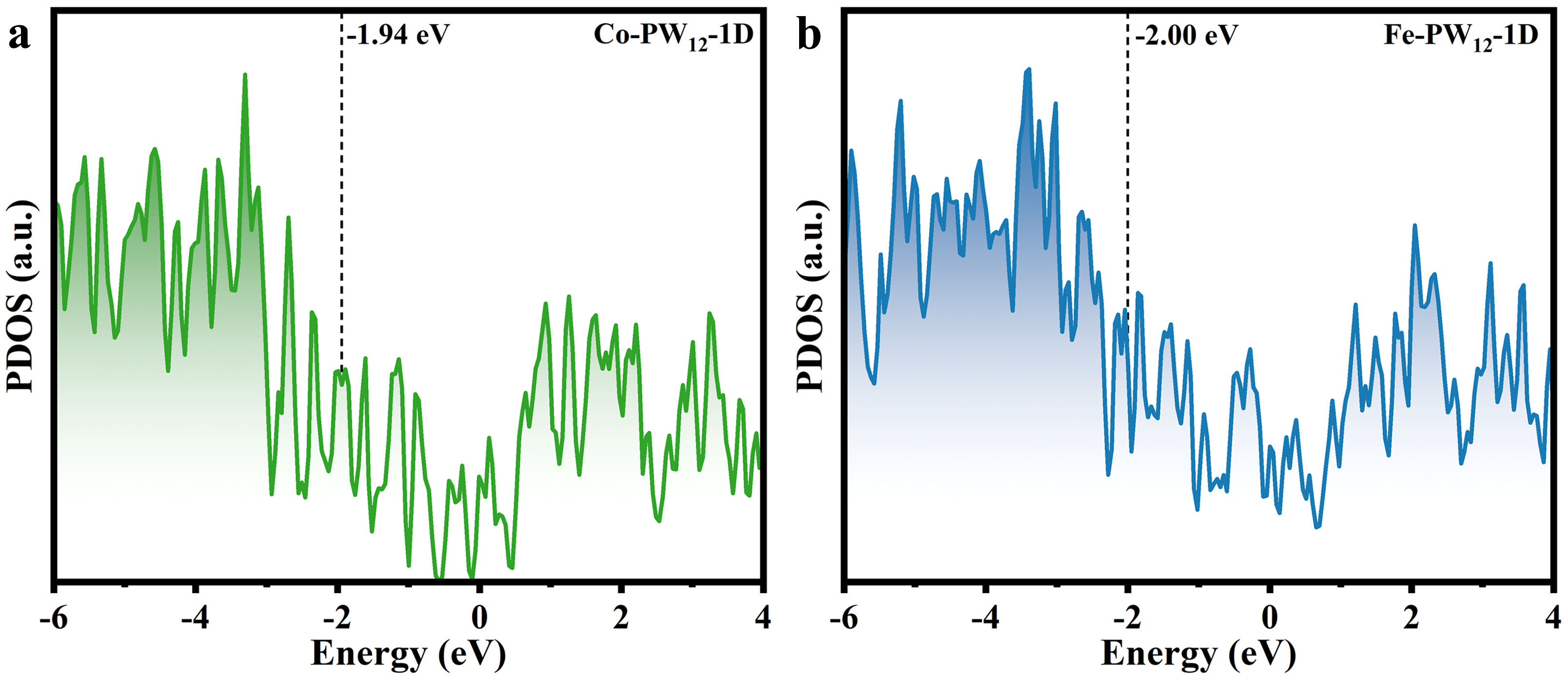


**Figure S28.** Projected density of states of (a) Co-PW_12_-1D and (b) Fe-PW_12_-1D, respectively.

**Table S1.** Crystal data and structure refinements for Ni-PW_12_-1D, Ni-SiW_12_-1D and Co-SiW_12_-1D.

|  | Ni-PW_12_-1D | Ni-SiW_12_-1D | Co-SiW_12_-1D |
| --- | --- | --- | --- |
| formula | C_80_H_60_N_16_Ni_6_O_60_PW_12_ | C_80_H_60_N_16_Ni_4_O_56_SiW_12_ | C_80_H_60_N_16_Co_4_O_56_SiW_12_ |
| Formula weight | 4794.87 | 4610.57 | 4611.45 |
| Crystal system | monoclinic | monoclinic | monoclinic |
| space group | C2/m | C2/m | C2/m |
| a (Å) | 14.9808(7) | 15.0914(5) | 15.1233(19) |
| b (Å) | 31.1873(17) | 30.8082(13) | 30.936(5) |
| c (Å) | 13.0483(6) | 12.9184(5) | 13.0057(17) |
| α (°) | 90 | 90 | 90 |
| β (°) | 95.706(2) | 95.7200(10) | 95.608(3) |
| γ (°) | 90 | 90 | 90 |
| V (Å^3^) | 6066.1(5) | 5976.4(4) | 6055.7(15) |
| Z | 2 | 2 | 2 |
| D_c_ (g cm^-3^) | 2.625 | 2.562 | 2.529 |
| μ (mm^-1^) | 12.339 | 12.211 | 11.977 |
| F(000) | 4406.0 | 4228.0 | 4220.0 |
| R_1_^a^[I > 2σ(I)] | 0.0708 | 0.0598 | 0.0591 |
| wR_2_^b^(all data) | 0.2148 | 0.1710 | 0.1839 |
| GOF on F^2^ | 1.049 | 1.013 | 1.037 |

^a)^R_1_ = ∑|F_o_| - |F_c_| / |F_o_|; ^b)^wR_2_ = {∑[w(F_o_^2^-F_c_^2^)^2^] / ∑[w(F_o_^2^)^2^]}^1/2^

**Table S2.** Selected bond distances (Å) and angles (°) for Ni-PW_12_-1D.

| **Atoms** | **Distance** | **Atoms** | **Distance** |
| --- | --- | --- | --- |
| Ni(1)-O(1)#4 | 2.048(10) | N(2)-O(2)#4 | 2.149(10) |
| Ni(1)-O(2)#5 | 2.226(11) | N(2)-O(2)#5 | 2.149(10) |
| Ni(1)-O(3) | 2.065(11) | N(2)-O(4) | 2.024(13) |
| Ni(1)-N(4) | 2.067(11) | N(2)-O(4)#6 | 2.024(13) |
| Ni(1)-O(4) | 2.057(11) | N(2)-O(5) | 1.813(13) |
| Ni(1)-N(3) | 2.057(12) | N(2)-C(5)#6 | 1.813(14) |
| **Atoms** | **Angle** | **Atoms** | **Angle** |
| O(1)#4-Ni(1)-O(2)#5 | 91.6(4) | O(2)#5-Ni(2)-O(2)#4 | 80.8(6) |
| O(1)#4-Ni(1)-O(3) | 90.0(4) | O(4)#6-Ni(2)-O(2)#5 | 90.6(4) |
| O(1)#4-Ni(1)-N(4) | 170.4(4) | O(4)-Ni(2)-O(2)#4 | 90.6(4) |
| O(1)#4-Ni(1)-O(4) | 89.5(4) | O(4)-Ni(2)-O(2)#5 | 80.4(4) |
| O(1)#4-Ni(1)-N(3) | 90.3(4) | O(4)#6-Ni(2)-O(2)#4 | 80.4(4) |
| O(3)-Ni(1)-O(2)#5 | 168.8(4) | O(4)-Ni(2)-O(4)#6 | 168.2(7) |
| O(3)-Ni(1)-N(4) | 90.5(5) | O(5)#6-Ni(2)-O(2)#5 | 89.9(5) |
| N(4)-Ni(1)-O(2)#5 | 89.7(4) | O(5)-Ni(2)-O(2)#4 | 89.9(5) |
| O(4)-Ni(1)-O(2)#5 | 77.9(5) | O(5)#6-Ni(2)-O(2)#4 | 167.5(6) |
| O(4)-Ni(1)-O(3) | 91.1(5) | O(5)-Ni(2)-O(2)#5 | 167.5(6) |
| O(4)-Ni(1)-N(4) | 100.0(5) | O(5)-Ni(2)-O(4) | 91.5(6) |
| N(3)-Ni(1)-O(2)#5 | 98.5(4) | O(5)#6-Ni(2)-O(4) | 96.0(6) |
| N(3)-Ni(1)-O(3) | 92.5(5) | O(5)#6-Ni(2)-O(4)#6 | 91.5(6) |
| N(3)-Ni(1)-N(4) | 80.2(5) | O(5)-Ni(2)-O(4)#6 | 96.0(6) |
| N(3)-Ni(1)-O(4) | 176.4(5) | O(5)#6-Ni(2)-O(5) | 100.5(10) |

Symmetry code: #1 = +x, 1-y, +z; #2 = 1-x, 1-y, 1-z; #3 = 1-x, +y, 1-z; #4 = -1/2+x, 1/2-y, -1+z; #5 = 3/2-x, 1/2-y, 1-z; #6 = 1-x, +y, -z.

**Table S3.** Selected bond distances (Å) and angles (°) for Ni-SiW_12_-1D.

| Atoms | Distance | Atoms | Angle |
| --- | --- | --- | --- |
| Ni(1)-O(3) | 2.043(6) | O(3)-Ni(1)-O(4)#4 | 96.7(3) |
| Ni(1)-O(4)#4 | 2.043(6) | O(3)-Ni(1)-O(2) | 88.0(3) |
| Ni(1)-O(2) | 2.092(7) | O(3)-Ni(1)-N(1)#5 | 88.7(3) |
| Ni(1)-N(1)#5 | 2.069(8) | O(3)-Ni(1)-O(1) | 95.5(3) |
| Ni(1)-O(1) | 2.049(7) | O(3)-Ni(1)-N(2)#5 | 167.9(3) |
| Ni(1)-N(2)#5 | 2.085(7) | O(2)-Ni(1)-O(4)#4 | 171.3(3) |
|  |  | N(1)#5-Ni(1)-O(4)#4 | 95.5(3) |
|  |  | N(1)#5-Ni(1)-O(2) | 91.9(3) |
|  |  | N(1)#5-Ni(1)-N(2)#5 | 79.7(3) |
|  |  | O(1)-Ni(1)-O(4)#4 | 84.3(3) |
|  |  | O(1)-Ni(1)-O(2) | 88.0(3) |
|  |  | O(1)-Ni(1)-N(1)#5 | 175.8(3) |
|  |  | O(1)-Ni(1)-N(2)#5 | 96.1(3) |
|  |  | N(2)#5-Ni(1)-O(4)#4 | 87.9(3) |
|  |  | N(2)#5-Ni(1)-O(2) | 88.8(3) |

Symmetry code: #1 = +x, 1-y, +z; #2 = 1-x, 1-y, 1-z; #3 = 1-x, +y, 1-z; #4 = 2-x, +y, 2+z; #5 = 1/2+x, 1/2-y, 1+z.

**Table S4.** Selected bond distances (Å) and angles (°) for Co-SiW_12_-1D.

| Atoms | Distance | Atoms | Angle |
| --- | --- | --- | --- |
| Co(1)-O(2)#4 | 2.167(9) | O(1)-Co(1)-O(2)#4 | 96.0(4) |
| Co(1)-O(1) | 2.062(9) | O(1)-Co(1)-O(4) | 90.3(4) |
| Co(1)-O(4) | 2.114(9) | O(1)-Co(1)-N(4)#5 | 166.3(4) |
| Co(1)-N(4)#5 | 2.134(10) | O(1)-Co(1)-N(3)#5 | 88.9(4) |
| Co(1)-N(3)#5 | 2.127(11) | O(1)-Co(1)-O(3) | 96.4(4) |
| Co(1)-O(3) | 2.093(10) | O(4)-Co(1)-O(2)#4 | 170.7(4) |
|  |  | O(4)-Co(1)-N(4)#5 | 88.2(4) |
|  |  | O(4)-Co(1)-N(3)#5 | 91.8(4) |
|  |  | N(4)#5-Co(1)-O(2)#4 | 87.2(4) |
|  |  | N(3)#5-Co(1)-O(2)#4 | 95.1(4) |
|  |  | N(3)#5-Co(1)-N(4)#5 | 77.6(4) |
|  |  | O(3)-Co(1)-O(2)#4 | 84.4(4) |
|  |  | O(3)-Co(1)-O(4) | 88.2(4) |
|  |  | O(3)-Co(1)-N(4)#5 | 97.1(4) |
|  |  | O(3)-Co(1)-N(3)#5 | 174.7(4) |

Symmetry code: #1 = +x, 1-y, +z; #2 = 1-x, +y, 1-z; #3 = 1-x, 1-y, 1-z; #4 = -x, +y, -z; #5 = -1/2+x, 3/2-y, -1+z.

**Table S5.** Fitting of the W 4f peak binding energies (eV) in PW_12_ and M-PW_12_-1D.

| Samples | W^VI^ | | W^V^ | |
| --- | --- | --- | --- | --- |
|  | W 4f_7/2_ | W 4f_5/2_ | W 4f_7/2_ | W 4f_5/2_ |
| Ni-PW_12_-1D | 36.1 | 38.3 | 35.6 | 37.8 |
| Co-PW_12_-1D | 36.0 | 38.1 | 35.5 | 37.6 |
| Fe-PW_12_-1D | 36.1 | 38.2 | 35.6 | 37.7 |
| Ni-SiW_12_-1D | 35.7 | 37.9 |  |  |
| Co-SiW_12_-1D | 35.7 | 37.9 |  |  |
| Fe-SiW_12_-1D | 35.8 | 37.9 |  |  |
| PW_12_ | 35.7 | 37.9 |  |  |
| SiW_12_ | 35.6 | 37.7 |  |  |

**Table S6.** The fitted resistance values of different catalysts.

| Catalyst | R_s_ (Ω) | R**_ct_** (Ω) |
| --- | --- | --- |
| Ni-PW_12_-1D | 11.9 | 129.7 |
| Co-PW_12_-1D | 12.7 | 141.2 |
| Fe-PW_12_-1D | 14.1 | 203.6 |
| Ni-SiW_12_-1D | 13.4 | 183.4 |
| Co-SiW_12_-1D | 14.4 | 211.9 |
| Fe-SiW_12_-1D | 14.6 | 258.1 |
| PW_12_ | 16.4 | 604.6 |
| SiW_12_ | 16.4 | 643.8 |

**Table S7.** Comparison of ENRA performance for some recently reported electrocatalysts in neutral electrolytes.

| Catalyst | Electrolyte | NO_3_^-^ Concentration | NH_3_ yield  (mg h^-1^ mg_cat._^-1^) | FE  (%) | Ref. |
| --- | --- | --- | --- | --- | --- |
| Cu_30%_@NHC | 0.1 M Na_2_SO_4_ | 0.1 M KNO_3_ | 3.63 | 77.05 | [7] |
| Pt-Fe_3_O_4_/NF | 0.1 M K_2_SO_4_ | 0.1 M KNO_3_ | 5.42 | 80.7 | [8] |
| Rh@Cu-0.6% | 0.1 M Na_2_SO_4_ | 0.1 M KNO_3_ | 2.54 | 93 | [9] |
| P_4_Mo_6_-based compound | 0.1 M Na_2_SO_4_ | 0.1 M NaNO_3_ | 8.97 | 89.65 | [10] |
| Rh NFs | 0.1 M Na_2_SO_4_ | 0.1 M KNO_3_ | 0.51 | 95 | [11] |
| CuNi-1 | 0.1 M Na_2_SO_4_ | 0.1 M KNO_3_ | 4.96 | 85.91 | [12] |
| Ni-P_4_Mo_6_/GO | 0.1 M Na_2_SO_4_ | 0.1 M KNO_3_ | 4.52 | 74.1 | [13] |
| CuNi/CP | 0.5 M Na_2_SO_4_ | 0.1 M NaNO_3_ | 3.53 | 90.36 | [14] |
| COF-366-M | 0.5 M Na_2_SO_4_ | 0.1 M KNO_3_ | 1.88 | 85.4 | [15] |
| Cu_2_Cl_2_(BINAP)_2_ | 0.5 M Na2SO4 | 0.1 M KNO_3_ | 6.35 | 94 | [16] |
| Pt_0.9_/Ce_0.5_-SS | 0.5 M Na_2_SO_4_ | 0.1 M KNO_3_ | 2.734 | 94.12 | [17] |
| NiPr-TPA-COF | 0.5 M K_2_SO_4_ | 0.1 M KNO_3_ | 2.5 | 90 | [18] |
| SiW_12_ | 0.1 M Na_2_SO_4_ | 0.1 M NaNO_3_ | 2.1 | 50.7 | This work |
| PW_12_ | 0.1 M Na_2_SO_4_ | 0.1 M NaNO_3_ | 2.7 | 51.0 | This work |
| Fe-SiW_12_-1D | 0.1 M Na_2_SO_4_ | 0.1 M NaNO_3_ | 6.2 | 67.4 | This work |
| Co-SiW_12_-1D | 0.1 M Na_2_SO_4_ | 0.1 M NaNO_3_ | 8.5 | 75.4 | This work |
| Ni-SiW_12_-1D | 0.1 M Na_2_SO_4_ | 0.1 M NaNO_3_ | 11.9 | 80.1 | This work |
| Fe-PW_12_-1D | 0.1 M Na_2_SO_4_ | 0.1 M NaNO_3_ | 9.2 | 79.4 | This work |
| Co-PW_12_-1D | 0.1 M Na_2_SO_4_ | 0.1 M NaNO_3_ | 12.5 | 84.7 | This work |
| Ni-PW_12_-1D | 0.1 M Na_2_SO_4_ | 0.1 M NaNO_3_ | 16.9 | 95.0 | This work |

References

[1] G. Kresse, J. Furthmuller, Efficient iterative schemes for ab initio total-energy calculations using a plane-wave basis set, *Phys. Rev. B.* **1996**, *54*, 11169.

[2] J. P. Perdew, K. Burke, M. Ernzerhof, Generalized gradient approximation made simple, *Phys. Rev. Lett.* **1996**, *77*, 3865.

[3] G. Kresse, J. Joubert, From ultrasoft pseudopotentials to the projector augmented-wave method, *Phys. Rev. B.* **1999**, *59*, 1758.

[4] P. E. Blöchl, Projector augmented-wave method, *Phys. Rev. B*. **1994**, *50*, 17953.

[5] S. Grimme, J. Antony, S. Ehrlich, H. Krieg, A consistent and accurate ab initio parametrization of density functional dispersion correction (DFT-D) for the 94 elements H-Pu, *J. Chem. Phys.* **2010**, *132*, 154104.

[6] G. Henkelman, B. P. Uberuaga, H. Jónsson, A climbing image nudged elastic band method for finding saddle points and minimum energy paths, *J. Chem. Phys.* **2000**, *113*, 9901.

[7] J. Zhang, C. Chen, R. Zhang, X. Wang, Y. Wei, M. Sun, Z. Liu, R. Ge, M. Ma, J. Tian, Size-induced d band center upshift of copper for efficient nitrate reduction to ammonia, *J. Colloid Interface Sci.* **2024**, *658*, 934.

[8] S. Mahmood, O. Alduhaish, M. Ammar, S. Khan, N. Ahmad, G. A. Ashraf, N. Afshan, N. Hassan, Pt-modified Fe_3_O_4_ supported on Ni foam nanocomposite for electrocatalytic nitrate reduction to ammonia, *Electrocatalysis* **2024**, *15*, 159.

[9] H. Liu, X. Lang, C. Zhu, J. Timoshenko, M. Rüscher, L. Bai, N. Guijarro, H. Yin, Y. Peng, J. Li, Z. Liu, W. Wang, B. R. Cuenya, J. Luo, Efficient electrochemical nitrate reduction to ammonia with copper-supported rhodium cluster and single-atom catalysts, *Angew. Chem. Int. Ed.* **2022**, *61*, e202202556.

[10] H. Luo, X. Wang, Q. Jiang, G. Li, X. Ren, H. Pang, H. Ma, Architecting cobalt-centered 2D sandwich-type polyoxometalate frameworks for selective electrocatalytic nitrate reduction to ammonia, Inorg. Chem. **2025**, *64*, 21054.

[11] H. Liu, J. Timoshenko, L. Bai, Q. Li, M. Rüscher, C. Sun, B. Roldan Cuenya, J. Luo, Low-coordination rhodium catalysts for an efficient electrochemical nitrate reduction to ammonia, *ACS Catal.* **2023**, *13*, 1513.

[12] L. Qi, Y. Fu, B. Ji, B. Sarsenbekuly, W. Kang, H. Yang, S. Liu, Bifunctional CuNi-x nano-alloys for electrocatalytic nitrate reduction and HPAM oxidation coupling reactions, *Mat. Chem. Front.* **2025**, *9*, 638.

[13] N. Zhao, X. Wang, S. Rong, Q. Jiang, H. Li, H. Pang, H. Ma, Polyoxometalate-based complex@graphene composite electrodes for efficient nitrate reduction to ammonia, *Dalton Trans.* **2025**, *54*, 9270.

[14] Q. Wang, Y. Shen, Electrocatalytic reduction of nitrate into ammonia by bimetallic copper‑nickel catalysts, *J. Electroanal. Chem.* **2025**, *987*, 119110.

[15] H. Hu, R. Miao, F. Yang, F. Duan, H. Zhu, Y. Hu, M. Du, S. Lu, Intrinsic activity of metalized porphyrin-based covalent organic frameworks for electrocatalytic nitrate reduction, *Adv. Energy Mater*. **2024**, *14*, 2302608.

[16] S. J. Zheng, X. Y. Dong, H. Chen, R. W. Huang, J. Cai, S. Q. Zang, Unveiling ionized interfacial water-induced localized H* enrichment for electrocatalytic nitrate reduction, *Angew. Chem. Int. Ed*. **2025**, *64*, e202413033.

[17] D. Chen, S. Zhang, D. Yin, W. Li, X. Bu, Q. Quan, Z. Lai, W. Wang, Y. Meng, C. Liu, S. Yip, F. R. Chen, C. Zhi, J. C. Ho, Tailored p-orbital delocalization by diatomic Pt-Ce induced interlayer spacing engineering for highly-efficient ammonia electrosynthesis, *Adv. Energy Mater*. **2023**, *13*, 2203201.

[18] F. Lv, M. Sun, Y. Hu, J. Xu, W. Huang, N. Han, B. Huang, Y. Li, Near-unity electrochemical conversion of nitrate to ammonia on crystalline nickel porphyrin-based covalent organic frameworks, *Energy Environ. Sci.* **2023**, *16*, 201.
